# Acute Low Alcohol Disrupts Hippocampus-Striatum Neural Correlate of Learning Strategy by Inhibition of PKA/CREB Pathway in Rats

**DOI:** 10.3389/fphar.2018.01439

**Published:** 2018-12-06

**Authors:** Wei Sun, Xiaoliang Li, Chunzhi Tang, Lei An

**Affiliations:** ^1^Medical College of Acupuncture-Moxibustion and Rehabilitation, Guangzhou University of Chinese Medicine, Guangzhou, China; ^2^College of Acupuncture-Moxibustion and Orthopedics, Guiyang University of Chinese Medicine, Guiyang, China; ^3^Department of Physiology, University of Saskatchewan, Saskatoon, SK, Canada

**Keywords:** acute alcohol, hippocampus, learning strategy, pCREB, neural oscillations

## Abstract

The hippocampus and striatum guide place-strategy and response-strategy learning, respectively, and they have dissociable roles in memory systems, which could compensate in case of temporary or permanent damage. Although acute alcohol (AA) treatment had been shown to have adverse effects on hippocampal function, whether it causes the functional compensation and the underlying mechanisms is unknown. In this study, rats treated with a low dose of AA avoided a hippocampus-dependent spatial strategy, instead preferring a striatum-dependent response strategy. Consistently, the learning-induced increase in hippocampal, but not striatal, pCREB was rendered less pronounced due to diminished activity of pPKA, but not pERK or pCaMKII. As rats approached the turn-decision area, Sp-cAMP, a PKA activator, was found to mitigate the inhibitory effect of AA on intra- and cross-structure synchronized neuronal oscillations, and rescue response-strategy bias and spatial learning deficits. Our study provides strong evidence of the critical link between neural couplings and strategy selection. Moreover, the PKA/CREB-signaling pathway is involved in the suppressive effect of AA on neural correlates of place-learning strategy. The novel important evidence provided here shows the functional couplings between the hippocampus and striatum in spatial learning processing and suggests possible avenues for therapeutic intervention.

## Introduction

Our memory is composed of multiple anatomically and functionally distinct systems (Squire and Zola, 1996). Two of these systems have received particular attention: a hippocampus- (HPC)-dependent system, which supports a place strategy, and a rather rigid, striatum- (DS)-dependent system, which supports a response strategy (Packard and Knowlton, 2002; Lee et al., 2008). Studies in rodents using maze paradigms to investigate the role of specific brain systems found that they are not independent but may interact in a cooperative (Kim and Baxter, 2001; Voermans et al., 2004) or competitive manner (Kim and Baxter, 2001; Poldrack et al., 2001).

Alcohol is one of the oldest and most widely consumed psychoactive drugs. The traditional view is that alcohol exposure affects the engagement of multiple memory systems (Matthews and Silvers, 2004; Gass et al., 2014). Previous studies with animal models have provided compelling evidence that alcohol can attenuate spatial memory mediated by the hippocampus with much greater potency than it attenuates learning, which is known to be mediated by other neural systems. For example, acutely administered alcohol has been shown to impair animals' ability to use previously acquired spatial memory, but not stimulus/response memory, suggesting selective impairment of spatial memory mechanisms that presumably depend on hippocampal function (Matthews et al., 1995). Using a dose-response analysis, they provided further evidence that alcohol attenuated the acquisition of spatial strategies to a greater extent than non-spatial strategies (White et al., 1998). Moreover, ethanol produces a shift in bias from the use of spatial information to non-spatial information to solve learning and memory tasks (Matthews et al., 1999). These findings clearly indicate that alcohol can disturb hippocampus-dependent “place” strategies but not “response” strategies. The neural mechanism underlying this alcohol-induced modification of multiple memory systems, however, remains elusive.

**Figure 1:**
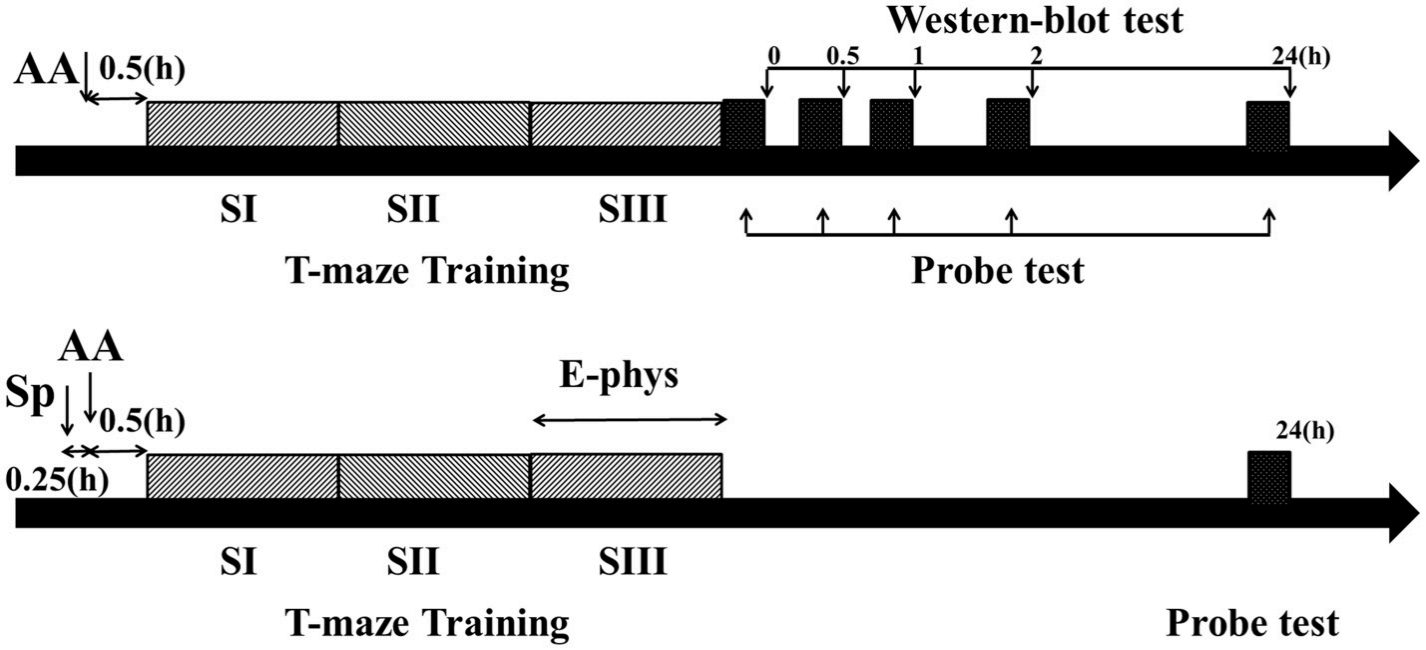
Schematic describing the experimental timelines. Timeline for behavior and Western blot experiments **(Top)**. Timeline for behavior and electrophysiological experiments **(Bottom)**. Dark gray bars showed three blocks training (each block consisted of five trials) and black bar showed probe test immediately (0 h), 0.5, 1, 2, or 24 h after training phase. AA, acute alcohol treatment; Sp, Sp-cAMP, a PKA activator, treatment; E-phys, electrophysiological recording during the third session of training day; Western blot test, Samples were collected and detected by Western bolt tests immediately, 0.5, 1, 2, or 24 h following T-maze learning.

One commonly used approach to study the process of memory formation is the T-maze reference paradigm for rodents (Watts et al., 2012; Machado et al., 2014). During the initial training, a food reward is located in one of two accessible arms of the maze. Rats are treated with alcohol acutely (AA) before initial training. To assess the effect of AA on the place/response strategy, the food reward is located in the same arm as previously, but rats start from the opposite testing arm in the probe test (Figure S1B). The HPC is important for context processing relevant to episodic memory. The importance of the temporal context is reflected in the dynamic relationship between cell firing (Moser et al., 2008) and the ongoing theta oscillation (Molter et al., 2012) and gamma oscillation (Kitanishi et al., 2015). The striatum is the largest nucleus of the basal ganglia, and its crucial role in reward processing, reward-mediated learning, and drug addiction is well established (Packard and Knowlton, 2002; Colombo et al., 2003; DeCoteau et al., 2007). Furthermore, the learning-related neural correlation between the hippocampus and striatum during acquisition of a spatial maze task has been reported recently (DeCoteau et al., 2007; Tort et al., 2008; van der Meer et al., 2010). Based on the above evidence, we asked whether neural activity in the hippocampus and striatum is different in terms of the response to place and response strategy, and how AA influences this process (Figure 1). Therefore, we recorded neural oscillations from multiple electrodes implanted in the hippocampus and striatum while animals performed the last five trials of the learning period in which they had already established the learning strategy after sufficient training. Functionally, distinct cognitive strategies were paralleled by brain-region-specific increases in the phosphorylation of the transcription factor cAMP response-element binding protein (pCREB) (Colombo et al., 2003; Carlezon et al., 2005). Pharmacological studies indicated that the blockade of ERK (extracellular signal-regulated kinase) (Sindreu et al., 2007; Seese et al., 2014), CaMKII/IV (calmodulin-dependent protein kinase II/IV) (Wang and Zhang, 2012), or PKA (cAMP-dependent protein kinase) signal pathway (Bernabeu et al., 1997; Vitolo et al., 2002) is accompanied by attenuated CREB activation and memory formation. In this regard, we further carried out a set of experiments in which the expressions of phosphorylated CREB, ERK, CaMKII, and PKA were detected. Finally, we employed pharmacological tools to rescue the expression of pCREB and shift the learning strategy induced by alcohol, and attempted to explore the underlying mechanism.

## Materials and Methods

### Animals and Drug Administration

Four hundred and seventy-nine male Sprague-Dawley rats (250–280 g; Beijing Research Center for Experimental Animals, Beijing, China) were individually housed and maintained on a 12-h light/12-h dark cycle (lights on at 0800) with free access to food and water. All experiments were performed at approximately the same time each day (between 1,300 and 1,700) and were approved by the Local Animal Care Committee. Animals were handled extensively (5 min each per day) for 2 weeks from the time of arrival to the first day of habituation. One week before maze testing, animals were reduced to 85% of their free-feeding weights over 7 d and maintained at this weight throughout the experiment. One day before habituation, animals received 0.5 g of chocolate chips (Milka; Kraft Foods) in addition to sufficient chow to maintain the 85% target weight and reduce neophobic reactions to chocolate during pre-training trials. During post-surgical recovery, animals were not food deprived till 1 day before they were re-trained. Since repeated exposure to alcohol may induce addiction and other effects on cognitive behaviors (Robinson et al., 2012; Jury et al., 2017), naive animals were used in the behavioral tasks, Western blot tests and electrophysiological recordings. Experimenters were blind to the treatment of the animals.

Analytical grade alcohol was purchased from Sinopharm Chemical Reagent Co., Ltd., Beijing, China. For acute alcohol intraperitoneal injection (i.p.), two previously established concentrations (20% v/v in saline; 1.0 or 2.0 g/kg), which were demonstrated to produce either spatial learning impairment (Rajendran and Spear, 2004) or cognitive impairment with locomotor deficits (Mitchell et al., 2011), were selected. Generally, studies of spatial learning and memory use low to high-doses of alcohol ranging from 1.0 to 2.5 g/kg alcohol (Matthews et al., 1999; Mitchell et al., 2011). Specifically, although there was no significant effect of acute alcohol exposure on reward choice in the risky decision-making task, a significant decrease in baseline locomotor activity at high-dose (1.5 g/kg) was found (Mitchell et al., 2011). Based on the studies reported by other groups, doses of at least 1.0 g/kg acute alcohol would inhibit hippocampal pyramidal neural activity either directly or indirectly (Tokunaga et al., 2003), resulting in behavioral disorders and hippocampal dysfunction in adult rats (White and Best, 2000). Additionally, the same range was also chosen by other labs to investigate the effect of acute alcohol on NR2B-NMDARs (Jones et al., 2010), which was associated with memory consolidation (Ge et al., 2010; Ghersi et al., 2015; An and Sun, 2018), and neural activity recording, which caused inhibitory effects on hippocampal CA1 pyramidal neurons (Givens, 1995; White and Best, 2000; Zhang et al., 2016). Because the dependent measure of most behavioral tests is the latency, changes in locomotor activity could be a confounding variable. Thus, a higher dose (2.0 g/kg) was chosen to compare with the dose of 1.0 g/kg in multiple spatial learning tests, since previous studies in which dose-response curves were examined demonstrated the reductions in locomotor activity (Stevenson et al., 2008; Mitchell et al., 2011). The time point of behavioral or recording initiation was chosen as previous findings, which indicated the concentration of alcohol in the striatum and hippocampus of awake rats reached a peak simultaneously at 30 min after dosing (Jamal et al., 2004; Lam and Gianoulakis, 2011). Matched controls receiving equivalent volumes of saline i.p. Alcohol and control injections were administered according to a pseudorandom design.

Sp-cAMP (Sp-Adenosine 3′,5′-cyclic monophosphorothioate triethylammonium salt hydrate; Sigma-Aldrich, St. Louis, MO, USA), a selective PKA activator, was dissolved in 0.1 M PBS (pH 6.9). Sp-cAMP was intracerebroventricular (i.c.v.) administrated (100 nmol/2 μL), bilateral infused into intra-hippocampal (intra-CA1; 0.21 or 2.1 nmol/0.5 μL) or intra-striatal (intra-DS; 0.21 nmol/0.5 μl) 15 min prior to an alcohol injection. The dose, time point and method of i.c.v. administration were chosen based on previous reports (Kumar et al., 2012; Gigante et al., 2014), which indicated efficiently regulated alcohol-induced behavioral changes. It should be noted that the 0.21-nmol dose is 10 times lower than the dose needed to impair working-memory performance, but sufficient to regulate CREB expression with non-specific changes in locomotor activity in rats (Ramos et al., 2006; Paine et al., 2009). Dosages were within the same range as used for other studies (Menezes et al., 2015; Dominguez et al., 2016).

There were 3 recording days (sessions) in the electrophysiological experiments. Each recording session was conducted as shown in Figure 1-bottom. Each rat that used place-strategy or response-strategy was randomly selected and received a treatment on each recording day. There were at least 5 drug-free days between the recording days. In the other behavioral or Western blot tests, each rat was only received one treatment.

### Behavioral Apparatus and Pre-training

Rats were trained according to the versions of the T-maze task with modification (DeCoteau et al., 2007; Tort et al., 2008; Schreiweis et al., 2014). Pre-training was conducted in a black Plexiglas cross maze consisting of 2 start arms (e.g., north and south arm) and 2 test arms (e.g., east and west arm). All 4 arms were 45 cm long, 14-cm width, 8-cm height. Arms diverged at a 90° angle from each other. Only one start arm was used during the pre-training days, the other start arm was blocked by removable black Plexiglas barrier. Experimental room contained various distal spatial cues. At the beginning of a trial, the rat was placed in a start Plexiglas box (18 × 14 × 14) with a blocker which allowed rat entered the start arm. Small brown bowl (4.5 cm in height, 9 cm in diameter) 1 cm before the end of the arms prevented visual inspection for food presence from a distance. Rat was allowed to visit all available maze arms until it obtained reward. If a rat did not reach the reward within 3 min, it was placed in the inter-trial Plexiglas box (45 × 45 × 52). After reward consumption, the experimenter placed the rat in the inter-trial box for 10–20 s. After each trial, arms were cleaned with 70% alcohol and allowed to dry completely, before the goal arm was re-baited. The reward location was fixed but changed in a pseudo-random order during each training day. Rats were habituated for 3 d with 5 trials per day. On the fourth day, 3 sessions (5 trials per session) was applied with 5 min interval. Rats were trained every 3 days until 15 trials choice accuracy was 90% or above. Then, rats designated to electrophysiology experiments were implanted.

### Strategy Learning and Probe Test

Following post-surgical recovery, rats were re-trained on the maze. During the first three sessions (training sessions, including session I, II, and III) each rat ran the task on which it had been originally trained. Then the rat was placed in the inter-trial room with the same dark/light schedule as before for 24 h during which time rat free accessed to water. One additional session (5 trials per session) was conducted on the Second day. During the fourth session (probe session, session IV), the first two trails were the conducted as session I, while during the third trial rats were subjected to a probe trial to assess the learning strategy used during the training. During the probe trial, the animals started from the opposite testing arm while the original start arm was blocked. The original or opposite start arm was randomly selected using a counterbalanced design in the following fourth and fifth trials. Rats were rewarded whatever the choice they made in probe test. The same maze and environment were used for all sessions, thereby insuring common sensory, behavioral, and motivational experiences. The probe trial had two possible outcomes: (1) rats using a place (spatial) strategy would visit the arm that was baited during training, i.e., the same spatial location (Figure S1B, top) or (2) rats using a response strategy would make the same turn as they had done during training and would visit the other arm (Figure S1B, bottom). This task required continuous re-learning and retrieving the learned strategy. Rats were trained every 4 days till a criterion (14 out of 15 correct choices) reached in 5 consecutive training days. Four days after training sessions, the Western blot (Figure 1-top) test or recording (Figure 1-bottom) was performed. As shown in Figure 1-top, one probe session was conducted immediately, 0.5, 1, 2, or 24 h after necessary training trials or sessions during the Western blot tests. Additionally, to ensure the effect of alcohol on leaning strategy, the probe session was conducted immediately after training phase (for example, after 5 or 15 training trails in **Figures 3F,G**). One day after recording, the probe session was also conducted to ensure the strategy did not switch (Figure 1-bottom). None exchange was found on the day following the neural activity recording.

To test whether acute alcohol (1.0 or 2.0 g/kg) causes a shift in the learning strategy, rats were treated with alcohol 30 min before training initiation. Then rats were subjected to three training sessions to acquire the learning strategy and one probe session to assess the learning strategy. To rescue the shift learning strategy induced by acute alcohol (1.0 g/kg), Sp-cAMP was i.c.v. administrated (100 nmol/2 μL), or bilateral infused (0.21 or 2.1 nmol/0.5 μl) 15 min prior to alcohol (**Figure 4A**).

### Electrophysiology

Microelectrode array (4 tetrodes) were arranged in two 2 by 2 matrix using 25-μm-diameter platinum/iridium wire, coated with polyimide (California Fine Wire Company) in a 32-gauge silica tube (World Precision Instruments). It was then attached via gold pins to an EIB-36-PTB board (Neuralynx Inc.), which was assembled to microdrive (Harlan 8-drive; Neuralynx). The electrode tips were gold-plated to maintain the impedance to 200–600 kΩ measured at 1 kHz. Electrodes were checked daily for spontaneous cellular activity via a pre-amplification headstage (NB labs, Denison, TX; sampling frequency of 26–32 kHz).

Rats were anesthetized with isoflurane and prepared for surgery using previously reported procedures (An et al., 2018; Sun et al., 2018). Two electrode arrays were chronically implanted: one above dorsal CA1 region of the hippocampus (AP: −3.3 mm, ML: ±2.2 mm, DV: 2.4–2.8 mm; HPC) and the other one above dorsal striatum (AP: +0.5 mm, ML: ±3.0 mm, DV: 3.6–4.6 mm; DS) in the ipsilateral hemisphere. The left or right hemisphere was implanted randomly but counterbalanced across rats. A stainless steel wire was used as ground electrode and soldered onto one jewelers' screw, which was implanted into the skull. The electrode array was fastened to the cranium by dental acrylic with additional scull screws as anchors.

Electrodes were advanced by, typically, 40 μm, the tissue was allowed to relax overnight. To be accepted for recording, cell signals had to be at least three times greater than the background activity. Recording sessions began when single unit activity was well isolated on hippocampus and/or striatum recording electrodes. If the unit signal(s) remained the next day the electrodes were lowered further in order to sample new neurons.

**Figure 2:**
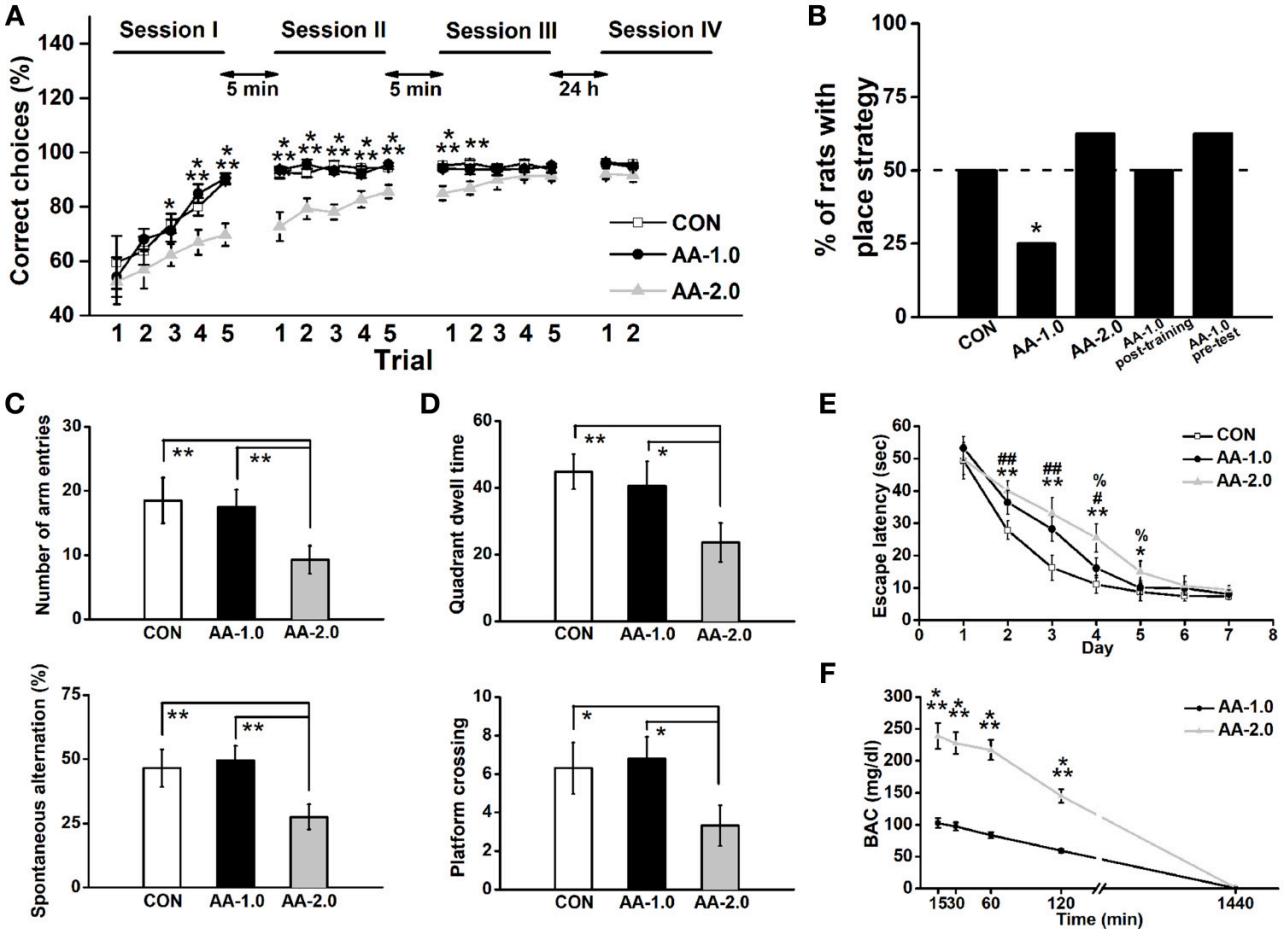
Low dose of AA induced response-strategy bias. Rats were i.p. injected with 1.0 g/kg (AA-1.0) or 2.0 g/kg (AA-2.0) alcohol 30 min before learning. **(A)** After 3 sessions of training, the performance of both control (CON) and low dose of AA (1.0 g/kg; AA-1.0) groups improved more rapidly than high dose of AA (2.0 g/kg; AA-2.0) group. During two trials of the session IV (probe session) was performed on the Second day, no difference among groups was found. (^*^P < 0.05, CON, or AA-1.0 vs. AA-2.0; ^**^P < 0.01, CON, or AA-1.0 vs. AA-2.0; ^***^P < 0.001, CON, or AA-1.0 vs. AA-2.0). *n* = 8 for each group. **(B)** The percentage of animals that used a place strategy in last three trials of probe test, during which rats started from the opposite start arm (the start arm that was used during learning was blocked). Low dose group preferred to use a response strategy. (^*^P < 0.05 vs. only CON). *n* = 8 for each group. **(C)** In the Y-maze task, the number of arm entries (top) and percentage spontaneous alternation (bottom) were similar between low dose alcohol and control group. However, high dose of AA group made significantly low entries and percentage alternation. (^**^P < 0.01). *n* = 8 for each group. **(D)** After acquisition the location of platform, high but not low dose of alcohol induced low quadrant dwell time (top) and the number of platform crossing (bottom). (^*^P < 0.05; ^**^P < 0.01). *n* = 8 for each group. **(E)** During the 7-day hidden-platform training, escape latency of both alcohol groups were prolonged but similar to control group in the last 2 training days. (^*^P < 0.05, CON vs. AA-2.0; ^**^P < 0.01, CON vs. AA-2.0; #*P* < 0.05, CON vs. AA-1.0; ##*P* < 0.01, CON vs. AA-1.0; %*P* < 0.05, AA-1.0 vs. AA-2.0). *n* = 8 for each group. **(F)** BACs were detected following low or high dose of alcohol treatment. Both treatments enhanced BAC concentration, with a much higher level in AA-2.0 group. (^***^P < 0.001, AA-1.0 vs. AA-2.0). *n* = 5 for each group.

The recording was performance with a Digital Cheetah system (Cheetah software, Neuralynx Inc.). Unit signals were recorded via a HS-36 unit gain headstage (Neuralynx Inc.) mounted on animal's head by means of lightweight cabling that passed through a commutator (Neuralynx Inc.), which allowed the animal to move unrestricted. Unit activity was amplified (1,000–10,000 times), sampled at 32 kHz and 600–6,000 Hz band-pass filters (Figure S4). LFPs were sampled at 32 kHz and filtered at 0.1–9,000 Hz from each electrode (Figure S5). In the experimental room, neural signals were transferred through a slip-ring commutator (Neuralynx) to the data acquisition system. To verify the stability of recording, unit activities were recorded for about 15 min before the training initiation. Only the performance on session III was recorded, since the behavioral strategy was formed successfully after appropriate training (Figure 2A). The animals' behavior was monitored by a digital ceiling camera (Neuralynx Inc.) and sand the CCD camera's signal was fed to a frame grabber (sampling rate, 1 MHz) with the experimental time superimposed for offline analysis. A careful slow motion video analysis of behavior was conducted to ensure that each timestamp of the task or delay-specific related event with respect to concurrent spike activity was precisely scored.

Observers scored each recording trials for the each event happened (microseconds) of the following behaviors (Figure S1A): Trial start (running out of the plastic box when hind-paws cross the door); Turn start (fore-paws entering the center area); Turn end (hind-paws leaving the center area); Reward (head entering reward bowl).

After the completion of all recording sessions, selective positions of individual electrodes were marked by electrolytic lesions (10 μA current for 10 s). Rats were sacrificed by urethane overdose and perfused transcardially with saline followed by 10% formalin. Brains were removed and post-fixed in a 10% formalin-10% sucrose solution. Brains were sectioned and recording sites were identified using standard protocols with reference to The Rat Brain in Stereotaxic Coordinates (1997, third edition). Only data from rats with probes contained within both hippocampus and striatum were collected (Figure S1C). Only when rats entered the same test arm during the each probe session we confirmed that the animal used the strategy during the training phase. Indeed, each rat persistently used the same strategy during the electrophysiological recordings.

In electrophysiological recording, the rats that used place-strategy or response-strategy were randomly divided into three treatments on each recording day (**Figures 5**, 1-bottom): (1) in CONP-Sp or CONR-Sp group, rats were i.c.v. administrated with Sp-cAMP (100 nmol/2 μL) 30 min before training sessions; (2) in AAP or AAR group, rats were treated with acute alcohol (1.0 g/kg) 30 min before training sessions; (2) in AAP-Sp or AAR-Sp group, rats were i.c.v. administrated with Sp-cAMP (100 nmol/2 μL) 15 min prior to alcohol.

### Directional Unit Phase-Locking Analysis

All calculations and statistical tests were done with Matlab (MathWorks) using custom written Matlab scripts (Berens, 2009). The analyses period was turn start till turn end. LFPs, which were recorded simultaneously in the same (intra-region analysis) or the two other brain areas (across region analysis), were used. The unit was recorded on a different tetrode than the LFP to avoid a possible frequency bias when the LFP around a spike was filtered. Each spike was assigned a phase derived from Hilbert transformations of respective band-pass-filtered LFPs (bands as above). The mean value of vector length was calculated as a measure of phase-locking strength and significance was determined by Rayleigh's test for circular uniformity. To determine directionality of the significantly phase-locked units, phase histograms (90° per bin) was built for each single unit spike train in relation to the theta filtered LFP. Directionality was determined by the location of the peak phase value for cells.

### Spectral Coherence Analysis

Spectral analysis of coherence was performed by using Neuroexplorer. Coherence measures the correlation between two signals as a function of frequency (Pereda et al., 2005). Coherence between two signals is calculated by the ratio of the cross-spectrum (between two channels) to the auto-spectrum (within channel). Coherence analysis between LFPs from two regions was performed using 1024 frequencies between 1 and 200 Hz. The overlap window was set at 5%, and it was smoothed with a Gaussian kernel with a bin-with of 3. Coherence values ranged from 0 to 1, where a value of 1 represents a perfect synchrony between signals for a given frequency. The peak coherence values within each band were averaged across all recordings for each animal, providing a mean coherence value for each animal within each frequency band.

### The Phase-to-Amplitude Coupling Algorithm

To quantify the amplitude modulation by phase, we created Tort's modulation index (MI) based on a normalized entropy measure previously described (Tort et al., 2008; Voloh et al., 2015). Briefly, the preprocessed voltage trace from a single trial, *x*_*raw*_*(t)* (raw signal), was filtered into two frequency bands of interest *f*_*P*_ and *f*_*A*_, generating *x*_*fP*_*(t)* and *x*_*fA*_*(t)*, respectively. The standard Hilbert transform was applied to extract the time series of the phase ϕ_*fP*_*(t)* and amplitude envelope *A*_*fA*_*(t)* from *x*_*fP*_*(t)* and *x*_*fA*_*(t)*, respectively. The composite time series [ϕ_*fP*_*(t), A*_*fA*_*(t)*] were then constructed, which caculated the amplitude of the *f*_*A*_ phase onto each phase of the *f*_*P*_ rhythm. The phase ϕ_*fP*_*(t)* were binned into interval of phase (*n* = 18; 20 intervals) (Tort et al., 2008; Voloh et al., 2015). The mean amplitude (*A*_*fA*_)_φ_*fp*__(*j*) of *A*_*fA*_*(j)* was computed over each phase bin *j*. The entropy measure *H* was calculated as follows:

(1) $$H=-\sum_{j=1}^{N}\frac{{\left(A_{fA}\right)}_{\varphi_{fp}}(j)}{\sum_{j=1}^{N}{\left(A_{fA}\right)}_{\varphi_{fp}}(j)}\cdot \text{log}\frac{{\left(A_{fA}\right)}_{\varphi_{fp}}(j)}{\sum_{j=1}^{N}{\left(A_{fA}\right)}_{\varphi_{fp}}(j)}$$

Where *N* = 18, which was equal to the number of bins.

The null hypothesis of the test is that the expected amplitude distribution is uniform. Thus, the MI is the normalized H by the uniform distribution (log*(N)*),

(2) $$MI=\frac{\text{log}(N)-H}{\text{log}(N)}$$

The range of MI value was larger than zero. A value above zero indicated the phase-to-amplitude modulation. The stronger phase-to-amplitude modulation, the larger MI value would be.

### Bilateral and Intracerebroventricular Microinjection

Rats were anesthetized with isoflurane and prepared for surgery as above. By using standard stereotaxic frame (SN-3, Narishige, Japan), stainless steel guide cannulae (22 gauge; Plastics One, Inc.) were bilaterally inserted above the HPC and the DS. A sterile stainless steel stylet (30-gauge, 10 mm, Plastics One Inc.) was inserted into guide cannula to avoid obstruction. For the i.c.v. infusion, a guide cannulae (22 gauge; Plastics One, Inc.) was unilaterally targeted to a lateral ventricle (AP: −0.8 mm, ML: ±1.2 mm, DV: 2.5 mm). Ventricular administration of compounds has been shown to quickly penetrate multiple brain regions, including hippocampal (Bai et al., 2016; Uhrig et al., 2017) and striatal areas (Marti et al., 2012; Cunha et al., 2017). Rats were given a minimum of 7 days to recover.

Infusions were achieved by inserting 30-gauge pacifier needles (10 mm, Small Parts Inc.) linked via PE-50 tubing to a microsyringe pump (Harvard Apparatus), extended 1.0 mm past the end of the cannulae. Habituation sessions were conducted four times for each rat in the week preceding formal experimentation.

Needles were inserted into both cannula and then delivery of Sp-cAMP or the vehicle into the HPC or the DS area was infused (0.5 μl/min). The infusion needles were left in place for 5 min after the infusion to permit diffusion of the drug. Rats were trained on the T-maze 30 min following infusions. Sp-cAMP (0.21 or 2.1 nmol/0.5 μL/side) and vehicle (0.1 M PBS) were given in a counterbalanced order. The same procedure was conducted in the i.c.v. injection (100 nmol/2 μL), but unilaterally infused into lateral ventricle.

Following testing in all conditions, the rats were sacrificed with urethane and the brain was collected as above. The placements of cannulae were determined. Only data obtained from rats with correctly inserted needles were included in statistical analysis.

### Protein Preparations and Western Blot Analysis

To assess whether changes in learning strategy after alcohol exposure were associated with changes in regional brain activity, we performed immunohistochemistry for the transcription factor pCREB, a critical element in memory formation (Carlezon et al., 2005; Chen et al., 2010). The expression of phosphorylated ERK (pERK), CaMKII (pCaMKII), and PKA (pPKA) were also detected to explore which upstream pathway or pathways involved in the alteration induced by alcohol (1.0 g/kg).

Additional groups of rats were trained and killed by overdose of urethane immediately, 0.5, 1, 2, or 24 h following strategy learning (Figure 1-top). Dorsal hippocampi and striatum were dissected in ice-cold cortical dissection buffer and snap-frozen in liquid N_2_ and stored at −80°C. Tissues was homogenized in ice-cold lysis buffer (pH 7.4) containing a cocktail of protein phosphatase and proteinase inhibitors (Sigma) to avoid dephosphorylation and degradation of proteins. The protein concentration of the supernatant was determined by BCA protein assay kit. Twenty micrograms (15 μL) of total protein per lane were resolved in 10% SDS-PAGE gels followed by electro-transferring to PVDF membranes (Pall, Pensacola, FL). Non-specific binding of antibodies to membranes was blocked with 5% (w/v) non-fat milk for 1 h at room temperature and incubated at 4°C overnight with a diluted primary antibody: rabbit anti-CREB (1:1,000; Santa Cruz Biotechnology, Santa Cruz, CA), rabbit anti-pCREB (1:1,000; Cell Signaling Technology, Beverly, MA), rabbit anti-pERK1/2 (1:2,000; Cell Signaling Technology, Beverly, MA), rabbit anti-CaMKII (1:1,000; Cell Signaling Technology, Beverly, MA), or rabbit anti-pPKA (1:1,000; Cell Signaling Technology, Beverly, MA). Mouse anti-β-actin (1:20,000; Sigma, MA) was used as an internal control. After washing in Tris-buffered saline-Tween 20 the membranes were incubated with horseradish-peroxidase (HRP)-conjugated secondary goat anti-mouse IgG (1:1,000) or goat anti-rabbit IgG (1:3,000) antibodies (Southern Biotechnology Associates, AL) incubated for 2 h at room temperature. The signal was detected by ECL Detection System following the manufacturer's instructions (Thermo Fisher Scientific, PA).

### Morris Water Maze (MWM) Task

Rats were tested in a tank 1.5 m in diameter and filled with 22°C water to a depth of 0.5 m. A circular platform (10-cm diameter) was used as the goal platform and distal extra-maze visual cues were surrounded. Rats were trained in standard version or modified version of MWM task. In the standard version, the task consisted of two phases: training phase and test phase. Briefly, during the training phase, animals were placed into the pool at start points that were pseudo-randomly selected, and the swim path to locate the submerged platform was tracked (Ethovision 2.0, Noldus, Wagenigen, Netherlands) for off-line analyzed. Rats were given 4 trials per day for 7 consecutive days till all groups were well-trained (no statistical difference among groups, Figure 2E). Test phase, during which the platform was taken out, were performed the following day. Rats were placed in the pool from a novel drop point. More details can be found in our previous studies (An and Sun, 2018). The purpose of this version was to compare the effect of alcohol on learning and memory with findings from other labs. In the modified version, rats were trained for three consecutive sessions (each session consisted of four trials) to find the platform with 5 min interval between sessions. The drop point was pseudo-randomly changed in the following trial but consistent in each trial for rats. Escape latency and Quadrant dwell time were measured. This 12-trial location learning test was applied to assess and compare with the results from T-maze, given that the similar endpoints were determined in two tasks.

### Y-maze

To mimic the testing conditions used in T-maze task, separated rats were selected and food restricted as above mentioned for 2 weeks after recovering from surgery. The maze was consisted of three arms (40 cm × 15-cm × 8-cm) separated with 120° angles and built of black Plexiglas. The procedure was conducted as a previous study (Mills et al., 2014). Briefly, rats were placed facing the wall at the end of one randomly selected arm as start arm, and allowed to explore the maze for 8 min. Number of entries to each arm and sequence of entries were recorded. A successful series of alternation entries was scored when the rats sequentially visited each arm (i.e., ABC or CBA, but not ABA). Percentage of spontaneous alternation was then calculated as actual alternations divided by total possible alternations.

### Locomotor Activity

Locomotor activity of the adult rats treated with alcohol (i.p.; 1.0 or 2.0 g/kg), Sp-cAMP (intraCA1; 0.21 or 2.1 nmol), or saline (i.p.) was measured (distance traveled, cm) in a Plexiglas chamber (53 × 58 × 43 cm;, Columbus Instruments, Columbus, OH) assisted by VersaMax Activity Monitoring System (AccuScan Instruments, Columbus, OH) as described previously (Hryhorczuk et al., 2016). Briefly, rats were habituated to the chambers for 120 min on day 1, without administration of any drug. On the second day, rats were treated with drug and immediately returned to chamber for a 120-min test.

### Blood Alcohol Concentration

Following i.p. injected with 1.0 or 2.0 g/kg alcohol, rat's tail was nicked and blood collected at 5 different time point (15, 30, 60, 120, or 1,440 min). Blood alcohol concentration (BAC) was quantified by using the nicotinamide adenine dinucleotide (NAD)-NADH enzymatic assay kit (Sigma Chemical, St. Louis, MO).

### Statistical Analysis

Data are expressed as mean ± S.E.M. All analyses were performed with Neuroexplorer, Matlab (MathWorks) and SPSS 17.0 software, which analyzed with one or two-way analysis of variance (ANOVA), multiple regression analysis, or Chi-square statistic, where appropriate. *Post-hoc* analysis with Turkey's test was conducted where appropriate. *P* < 0.05, *P* < 0.01, and *P* < 0.001 levels of confidence were used in the analyses.

## Results

### Acute Alcohol Induced a Learning Strategy Shift

Acute alcohol was i.p. injected to well-trained rats at either 1.0 g/kg (AA-1.0) or 2.0 g/kg (AA-2.0) dose 30 min before behavioral task. Controls (CON) received equivalent volumes of saline via i.p. All three groups of rats exhibited a chance level at the beginning of training session I, indicating that they had no preference for either of the two accessible arms (CON: 59.5 ± 9.7%, AA-1.0: 54.0 ± 7.3%, AA-1.0: 52.5 ± 8.3%; Figure 2A). The animals in three groups gradually learned to reach the reward arm [effect of trial: *F*_(14_, _294)_ = 37.93, *P* < 0.0001]. An ANOVA analysis also indicated significant effects of group [*F*_(2, 21)_ = 9.03, *P* < 0.001]. After 5 trials of learning, the CON and AA-1.0 groups reached correct ratio of 92.3 ± 1.9% and 93.8 ± 1.2%, respectively. However, the correct ratio of the AA-2.0 group reached 89.8 ± 3.5% till after the second trial of session III. Tukey's test indicated a significant effect of 2.0 g/kg alcohol exposure on learning the baited location during trial 3 (*P* < 0.05), 4 (*P* < 0.01), 5 (*P* < 0.001) of session I, trials of session II (*P* < 0.001 for all), trial 1 (*P* < 0.001), and 2 (*P* < 0.01) of session III. However, no significant effect of group [*F*_(2_, _21)_ = 2.39, *P* > 0.05] was observed during the first two trials of probe test that performed 24 h after learning. By blocking the arm opposite of the start arm, the maze was essentially used as a T maze to examine the strategy during learning period. Three groups of rats were tested 24 h after three learning sessions and the percentage of animals that used a place strategy was calculated by Chi-square test (discrete variables). The percentage of CON and AA-2.0 groups were nearly 50%, and the discrete variables of two groups were not significantly different (Chi-square test: *X*^2^ = 0.35, *df* = 1; *P* > 0.05, Figure 2B). There was a trend for a lower percentage of rats with place strategy in AA-1.0 group (Chi-square test: *X*^2^ = 4.95, *df* = 1; *P* < 0.05). When rats were injected with low-dose alcohol immediately post-training or 30 min before the probe test on the second day, the strategy preference was not observed (Chi-square test: *X*^2^ = 0.00, *df* = 1; *P* > 0.05 for AA-1.0 post-training; *X*^2^ = 0.35, *df* = 1; *P* > 0.05 for AA-1.0 pre-test).

Low-dose alcohol manifested strategy shifting in T-maze task was not associated with differences in exploratory motivation when rats were tested on a similar Y-maze apparatus, in this test animals showed similar arm entry number (*P* > 0.05, Figure 2C) and spontaneous alternation (*P* > 0.05) as CON group. However, compared with CON and AA-1.0 groups, high-dose alcohol impaired spatial working memory, manifested as significantly depressed incidences of both spontaneous performance (*P* < 0.01) and novel exploratory behavior (*P* < 0.01). More seriously, the high-dose alcohol disrupted spatial reference memory (Figure 2D), reduced quadrant dwell time (*P* < 0.01) and platform crossing number (*P* < 0.01) compared with CON or AA-1.0 group. However, these were not observed in AA-1.0 group. Nevertheless, prolonged escape latency was observed on training days in AA-1.0 group (day 2 and 3, *P* < 0.01; day 4, *P* < 0.05; Figure 2E), although their performance were better than AA-2.0 ones on day 4 (*P* < 0.05) and day 5 (*P* < 0.05). Rats of AA-2.0 group showed significantly prolonged escape latency to locate the platform (day 2, 3, and 4, *P* < 0.01; day 5, *P* < 0.05). Thus, both doses of alcohol disrupted the spatial learning process.

ANOVA conducted on BACs revealed effects of alcohol time [*F*_(4_, _32)_ = 61.88, *P* < 0.001; Figure 2F], and dose [*F*_(1_, _10)_ = 34.73, *P* < 0.001]. Tukey's test demonstrated the dose of 2.0 g/kg induced a higher BACs than 1.0 g/kg at 15, 30, 60, and 120 min (*P* < 0.001) after the treatment. However, this difference was not presented 24 h later. These results clearly indicated AA directly disrupted the performance during learning, but not memory or probe task that was conducted 24 h later.

Overall, low-dose alcohol undoubtedly impaired spatial learning ability (training phase of the MWM task). However, high-dose treatment disrupted both learning and spatial memory. Additionally, the locomotor ability was also impaired in AA-2.0 group. Given these evidence, it seemed plausible that AA-2.0 rats did not apply the previous strategy but randomly chose an arm. Therefore, we only employed the low-dose treatment in the following electrophysiological and Western blot tests.

### The Expression of CREB, pCREB, pERK, pPKA, and pCaMKII Following Training

**Figure 3:**
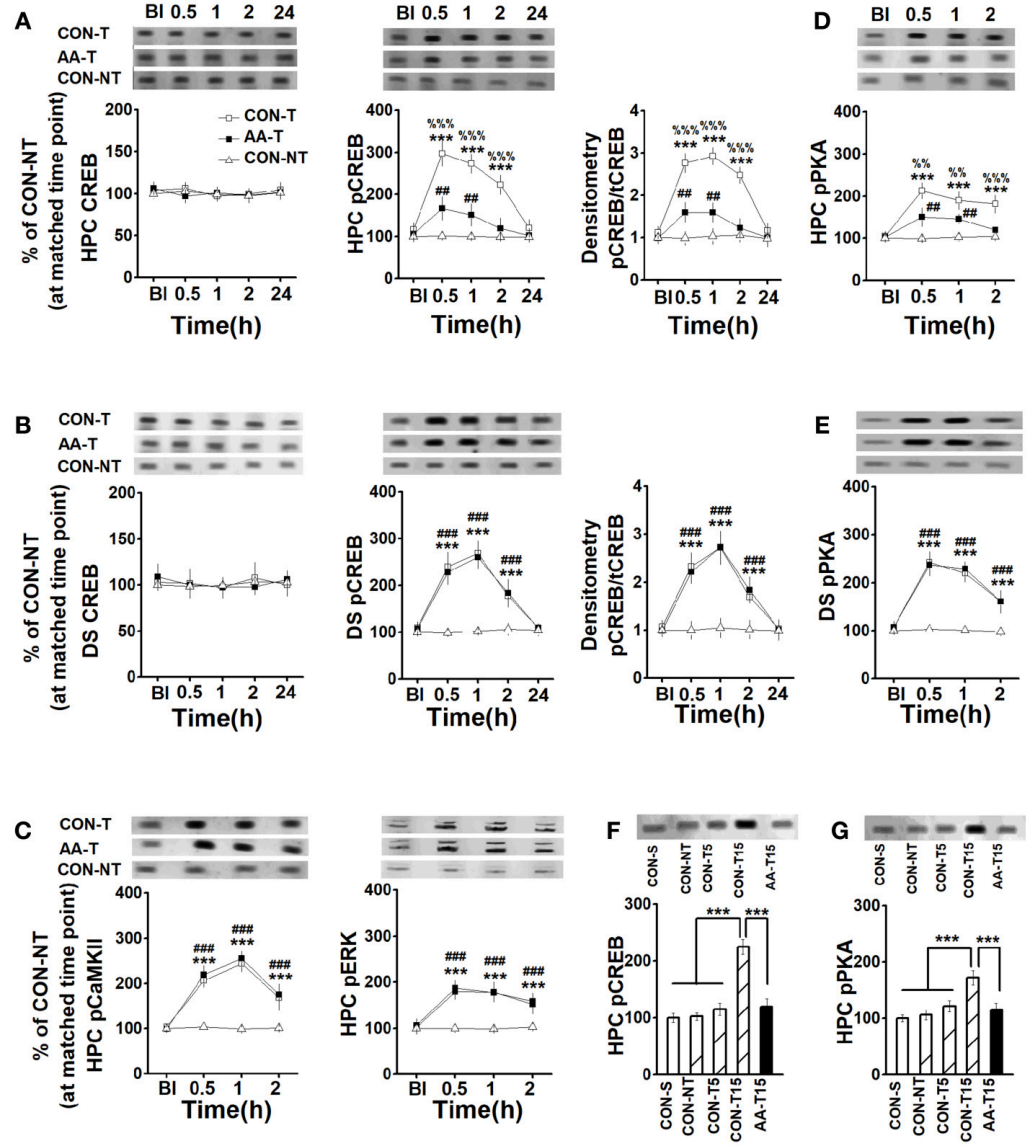
The expression of CREB, pCREB, pPKA, pCaMKII, and pERK in the HPC and DS following T-maze training. The expression of HPC CREB (left) and pCREB (middle), and the ratio of CREB to pCREB (right) **(A)**, pCaMKII (left), and pERK (right) **(C)**, pPKA **(D)** in rats that used place-strategy at different time points following training (Bl represented the time point of pre-learning). CON-T, AA-T, and CON-NT were represented the control training group, the AA training group and the control non-training group, respectively. *n* = 6 for each time point of each group. The expression of DS CREB (left) and pCREB (middle), and the ratio of CREB to pCREB **(B)**, and pPKA **(E)** in rats that used response-strategy at different time points following training. CON-T, AA-T, and CON-NT were represented the control training group, the AA training group and the control non-training group, respectively. *n* = 8 for each time point of each group. (^***^P < 0.001, CON-T vs. CON-NT; ##*P* < 0.01, ###*P* < 0.001, AA-T vs. CON-NT; %%*P* < 0.01, %%%*P* < 0.001, CON-T vs. AA-T). The expression of HPC pCREB **(F)** and pPKA **(G)** in rats that used place-strategy after different learning trials. Representative Western blots were shown in all above insets. CON-S, CON-NT, CON-T5, CON-T15, and AA-T15 were the represented saline home cage control group, the control non-training group, the control 5-trial training group, the control 15-trial training group and the AA 15-trial training group, respectively. (^***^P < 0.001). *n* = 5 for each group.

For total CREB expression, there were no main effects of time on the HPC [*F*_(4_, _84)_ = 0.27, *P* > 0.05, Figure 3A-left] or the DS [*F*_(4_, _60)_ = 0.39, *P* > 0.05, Figure 3B-left], or effects of group on the HPC [*F*_(2_, _15)_ = 0.32, *P* > 0.05] or the DS [*F*_(2_, _21)_ = 0.47, *P* > 0.05]. A two-way ANOVA analysis revealed a main effect of time in the HPC [*F*_(4_, _84)_ = 92.40, *P* < 0.001, Figure 3A-middle] and DS [*F*_(4_, _60)_ = 123.61, *P* < 0.001, Figure 3B-middle], a significant effect of group in the HPC [*F*_(2_, _15)_ = 89.54, *P* < 0.001] and DS [*F*_(2_, _21)_ = 63.31, *P* < 0.001] on pCREB expression. Three-session (15-trial) place-strategy training significantly enhanced the HPC pCREB levels of the alcohol (AA-T) (0.5, 1, and 2 h: *P* < 0.001) and control (CON-T) groups (0.5, 1, and 2 h: *P* < 0.001) compared to the cage control one (CON-NT). However, these elevated pCREB activity was lower in the AA-T group than the CON-T one (0.5 and 1 h: *P* < 0.01). More importantly, there was a main effect of time [*F*_(4_, _60)_ = 144.07, *P* < 0.001, Figure 3A-right], and group [*F*_(2_, _15)_ = 101.23, *P* < 0.001] on the ratio of pCREB to CREB in the HPC. Similarly, a main effect of time [*F*_(4_, _84)_ = 111.36, *P* < 0.001, Figure 3B-right] and group [*F*_(2_, _21)_ = 48.33, *P* < 0.001] were also found in the DS. Tukey's test indicated this ratio in the HPC was lower at 0.5 (*P* < 0.001) and 1.0 h (*P* < 0.001) post-training compared the AA-T group with CON-T group. However, we did not find these differences in DS. These results indicated alcohol could disrupt the activation of CREB but not its precursor. We sought to examine pCaMKII, pERK, and pPKA, expression to detect which pathway(s) involved in the reduction of pCREB level (Figures 3C,D). An ANOVA analysis indicated a significant effect of time [*F*_(4_, _60)_ = 162.17, *P* < 0.001] and group [*F*_(2_, _15)_ = 73.36, *P* < 0.001] were observed in pCaMKII expression. Similarly, a significant effect of time [*F*_(4_, _84)_ = 75.69, *P* < 0.001] and group [*F*_(2_, _21)_ = 54.99, *P* < 0.001] were observed on pERK expression. However, *post-hoc* analysis with Turkey's test found that AA-T did not differ significantly from the CON-T groups in pERK or pCaMKII expression. In the HPC, an ANOVA analysis indicated main effects of time [*F*_(4_, _60)_ = 66.30, *P* < 0.001; Figure 3D] and group [*F*_(2_, _15)_ = 64.04, *P* < 0.001] on pPKA level, with difference between the AA-T and CON-T groups (0.5, 1, and 2 h: *P* < 0.001). The main effect of time [*F*_(4_, _84)_ = 96.79, *P* < 0.001; Figure 3E] and group [*F*_(2_, _21)_ = 43.12, *P* < 0.001] were also found in pPKA of DS, but equivalent levels exhibited between the AA-T and CON-T groups. These results suggested the alcohol disrupted CREB phosphorylation via a pathway involving pPKA but not pERK or pCaMKII.

It should be emphasized that the expression of DS pCREB of place-strategy rats and the HPC pCREB level of response-strategy rats were also detected, but no statistical difference was found in the above proteins between the strategies (data not shown).

To confirm whether the enhanced pCREB and pPKA were regulated by the learning, we detected the levels of pCREB and pPKA following different learning trials, including 0 (CON-S), 5 (CON-T5), and 15 (CON-T15) (Figures 3F,G). An ANOVA analysis indicated a main effect of group in pCREB [*F*_(4_, _20)_ = 28.51, *P* < 0.001] and pPKA [*F*_(4_, _20)_ = 16.11, *P* < 0.001]. A similar level of pCREB was found in saline home cage control (CON-S) and environmental control (CON-NT), which was exposed to T-task maze for 2 min when CON-T15 (15-training-trial group) was training, as well the pPKA levels. These results indicated the environment (T-maze equipment) did not change pCREB and pPKA expression. Notably, statistical differences were found in CON-15 group relative to CON-S, CON-NT, and CON-T5 groups (*P* < 0.001, for all comparisons). However, even after 15 trials training, alcohol-treated group (AA-T15) still exhibited less expression of pCREB (*P* < 0.001) and pPKA (*P* < 0.001) than CON-T15 group.

### Reversal Effect of Sp-cAMP on AA-induced Behavioral Deficits and Neural Couplings

**Figure 4:**
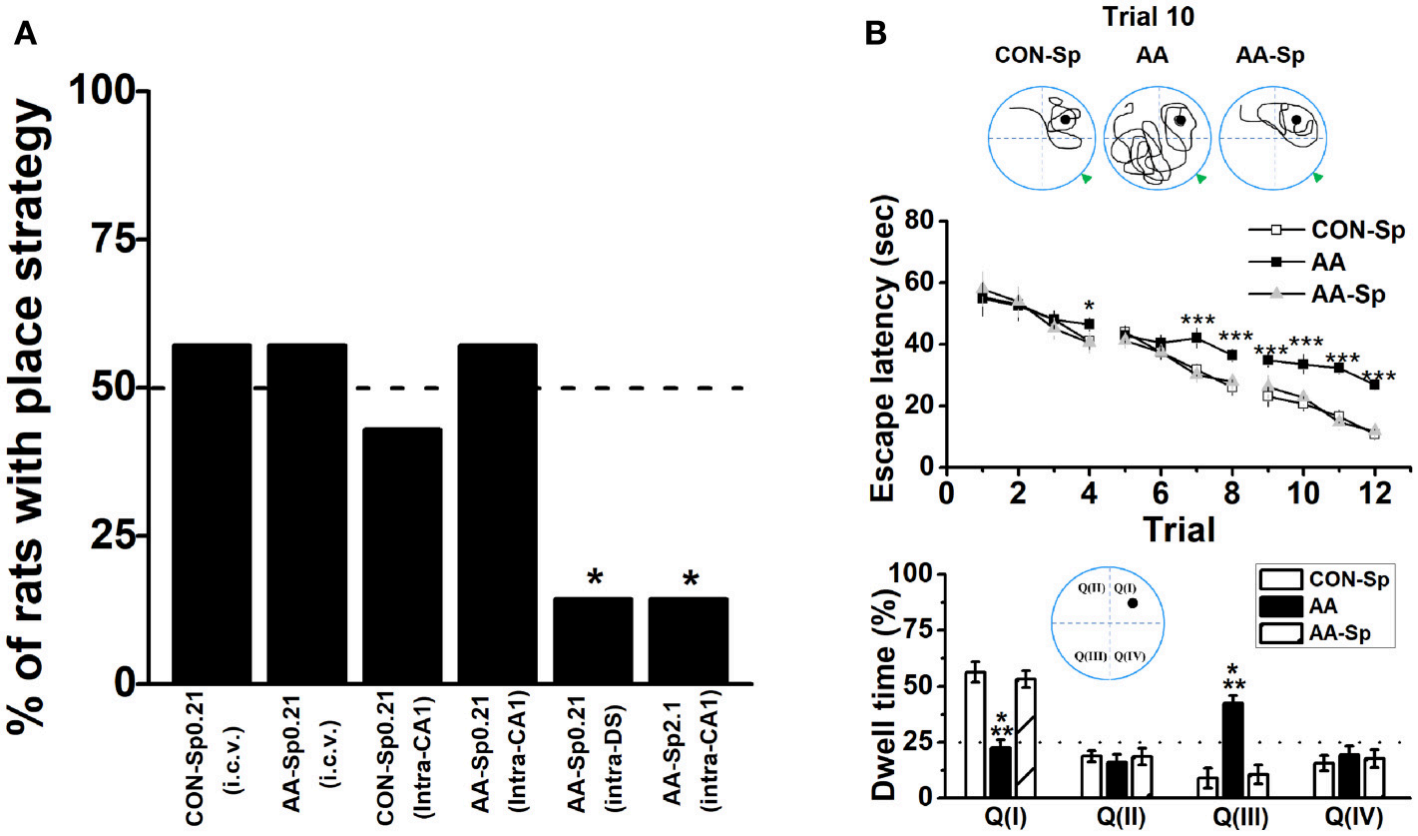
Infusion of Sp-cAMP into the HPC could mitigate the response-strategy bias and spatial learning deficits induced by AA. **(A)** The percentage of animals that used a place strategy. Intracerebroventricular (i.c.v.) infusion of Sp-cAMP into HPC (intra-CA1) or DS (intra-DS) was administrated to AA-induced rats at three different time points around Y-maze learning task. (^*^P < 0.05 vs. only AA-Sp0.21-intraCA1). *n* = 7 for each group. **(B)** Sp-cAMP reduced travel path during the 10th trial of location learning test (top). Traces of travel path following injection of the control (Sp-cAMP) (top-left), AA (top-middle), and AA combined with Sp-cAMP (top-right) were presented. The dark circles designate the position of the hidden platform and the gray triangle represented the starting position of the previous trial (trial 9). Escape latency (middle) and dwell time (bottom) were calculated for each trial during the 12-trial location learning task The distribution of quadrants in the MWM test (bottom insert). (^*^P < 0.05, ^***^P < 0.001, AA vs. CON-Sp or AA-Sp) *n* = 7 for each group.

Given alcohol-induced reduction of pCREB by blocking PKA phosphorylation, we administrated Sp-cAMP, a PKA activator, to enhance the activity of HPC pPKA and then rescue the response-strategy bias. As expected, when 0.21 nmol dose of Sp-cAMP was infused i.c.v. 15 min pre-AA treatment, rats did not exhibit strategy bias (Figure 4A). Furthermore, neither i.c.v. nor intra-CA1 Sp-cAMP infusion at this dose caused strategy preference in vehicle group, supporting the efficacy of Sp-cAMP in behavioral studies (Ramos et al., 2006; Paine et al., 2009). To test the region-specific effects of Sp-cAMP injection, Sp-cAMP was infused into the HPC (AA-Sp0.21-intraCA1) or the DS (AA-Sp0.21-intraDS). Compared to infusion into HPC (AA-Sp0.21-intraCA1), the infusion of Sp-cAMP into the DS (AA-Sp0.21-intraDS), significantly changed the percentage of rats with place strategy (Chi-square test: *X*^2^ = 6.35, *df* = 1; *P* < 0.05). Intriguingly, a higher dose of Sp-cAMP infusion into the HPC (AA-Sp2.1-intraCA1) significantly altered the proportion compared to AA-Sp0.21-intraCA1 group (Chi-square test: *X*^2^ = 6.35, *df* = 1; *P* < 0.05), which implied a bidirectional effect of Sp-cAMP.

Although either of the two available strategies were sufficient to locate the food reward during T-maze task, there was a particular difference between the systems underlying two strategies in terms of cognitive function. Therefore, the similar schedule applied in T-maze task was conducted in the 12-trial location learning MWM task, which was a well-validated method for examining hippocampus-dependent spatial learning ability (Vorhees and Williams, 2006). A repeated measures ANOVA analysis was conducted on escape latency confirmed statistical differences of group [*F*_(2_, _18)_ = 46.13, *P* < 0.001; Figure 4B-top] and trial [*F*_(11_, _198)_ = 33.66, *P* < 0.001], indicating effective learning over the training. Tukey's test revealed that the differences between the CON-Sp and AA groups were significant for escape latency (*P* < 0.001, for all comparisons). Similar results were shown between the AA-Sp and AA groups in the same training trial (*P* < 0.001, for all comparisons), while no difference was found between the CON-Sp and AA-Sp groups. Additionally, no main effect of group, trial or group by trial effect was found in swimming speed analysis (data not shown). In Figure 4B (bottom), there was a shortened trajectory in the trial 10 of learning stage in both the control group and the Sp-cAMP-treated alcohol group. However, more swimming trajectories in opposite but not target quadrant was strikingly observed in alcohol-treated rats. Observingly, in the trial 9, the sub-platform was on the right of drop point, while the target platform was on the left side in the trial 10. More importantly, the dwell time of the CON-Sp and the AA-Sp groups were less in Quadrant III (*P* < 0.001) but more in Quadrant I (*P* < 0.001) compared to AA group. No difference in the time spent in Quadrant II or Quadrant IV was detected among groups. The alcohol-impaired rats disregard the drop point or surrounding cues but tended to find the platform by an egocentric response strategy, implied alcohol impaired spatial learning ability, and lead rats to prefer response rather than place learning strategy.

**Figure 5:**
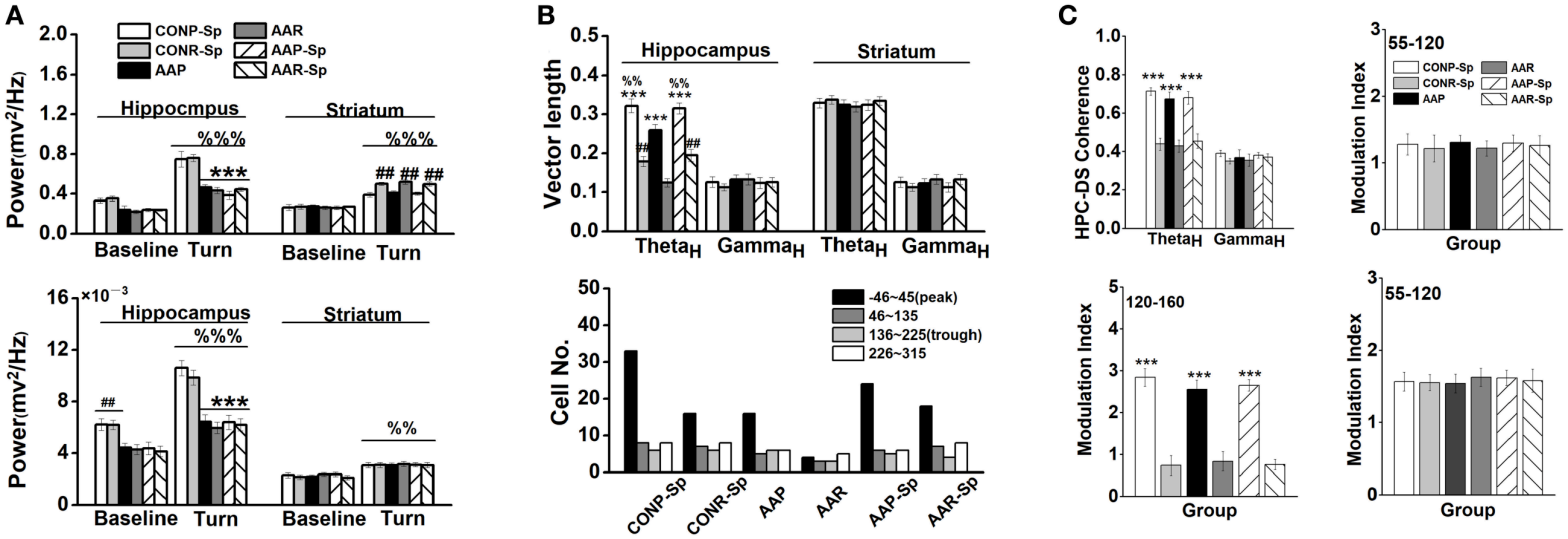
Infusion of Sp-cAMP reversed the functional couplings between the HPC and DS. **(A)** The power of HPC and DS high-theta oscillations (top), and the power of HPC and DS high-gamma oscillations during turn-decision period (bottom). (^***^P < 0.001, vs. CONP-Sp or CONR-Sp; ##*P* < 0.01, CONP-Sp vs. CONR-Sp, AAP vs. AAR, or AAP-Sp vs. AAR-Sp, %%*P* < 0.01, %%%*P* < 0.001, vs. matched baseline). *n* = 7 for each group. **(B)** The coupling of each spike on high-theta phase in the HPC and the DS. The vector length (top) and the number of unit (bottom) whose firings were coupled to high-theta oscillation in the HPC and DS during turn-decision period. (^***^P < 0.001, CONP-Sp vs. CONR-Sp, AAP vs. AAR, or AAP-Sp vs. AAR-Sp; ##*P* < 0.01, vs. AAR; %%*P* < 0.01, vs. AAP). *n* = 55 for CONP-Sp group; *n* = 37 for CONR-Sp group; *n* = 33 for AAP group; *n* = 15 for AAR group; *n* = 41 for AAP-Sp group; *n* = 37 for AAR-Sp group. *n* represented the number of neurons. **(C)** Modulation index (MI) phase-amplitude couplings between the DS high-theta phase and the amplitude of HPC high-gamma oscillations (top-right), between the DS high-theta phase and the amplitude of HPC high-frequency oscillations (bottom-left), and between the HPC high-theta phase and the amplitude of DS high-gamma oscillations (bottom-right) during tuning-decision period. (^***^P < 0.001, CONP-Sp vs. CONR-Sp, AAP vs. AAR, or AAP-Sp vs. AAR-Sp). *n* = 7 for each group.

Base on a similar effect of intra-CA1 and i.c.v. infusion of Sp-cAMP, we injected drug by i.c.v. infusion since the electrode probes and guide cannulae must be distantly implanted. The results that infused Sp-cAMP into the control groups were very similar with those of vehicle groups (Table S1). No significant effect of Sp-cAMP treatment on the percentage of animals with place strategy was found (Chi-square test: *X*^2^ = 0.13, *df* = 1; *P* > 0.05; CONP vs. CONP-Sp). Furthermore, no statistical difference was found in locomotor activity between CON and CON-Sp0.21 groups (Figure S3). Therefore, the comparison between the control and alcohol groups was not repeated if unnecessary. A two-way ANOVA analysis revealed a main effect of treatment in the HPC [*F*_(2_, _39)_ = 82.37, *P* < 0.001] on the high-theta oscillation during turn-decision period. Lower high-theta oscillation of Sp-cAMP-treated AA groups during turn-decision period were presented compared with control groups (*P* < 0.001, for all comparisons; Figure 5A-top). No significant difference was observed after Sp-cAMP infusion following alcohol exposure. A significant effect of strategy was found in the DS high-theta power [*F*_(1_, _39)_ = 23.30, *P* < 0.001; Figure 5A-top], which confirmed Sp-cAMP maintained the functional power to perform the response behavior. For high-gamma oscillation, the Sp-cAMP infusion didn't enhance the HPC low baseline or turn-related power, although it didn't affect the DS function (Figure 5A-bottom). An ANOVA analysis yielded a significant effect of strategy on the HPC vector length in high-theta band [*F*_(1_, _39)_ = 84.53, *P* < 0.001; Figure 5B top-left]. A two-way ANOVA analysis revealed a main effect of treatment in the HPC [*F*_(2_, _39)_ = 70.61, *P* < 0.001] on the phase-locking vector length of high-theta band. The HPC phase-locking vector length of alcohol group was extended by Sp-cAMP injection (*P* < 0.001, AAP-Sp vs. AAP). Strikingly, no statistical difference was found between the CONP-Sp and AAP-Sp groups. Again, no observably effect of Sp-cAMP was found in the DS (Figure 5B top-right), indicating the selective effect of Sp-cAMP on the HPC spike-phase locking in high-theta of both AA-treated strategy conditions. Figure 5B (bottom) shows that, except for alcohol-treated response group, the HPC neurons in other groups tended to phase-lock clearly to the peak of high-theta oscillation. The coherence between the HPC and DS of place strategy groups were strengthened in the range of the high-theta [*F*_(1_, _39)_ = 63.02, *P* < 0.001; Figure 5C top-left], but not high-gamma, around turn-decision area. The substantial DS theta-phase modulation of the HPC amplitude of oscillations in the gamma range during the turn-decision phase of the task in the place strategy conditions, confirmed by an ANOVA analysis [*F*_(1_, _39)_ = 141.89, *P* < 0.001; bottom-left]. No statistical difference was found in the MI of phase–amplitude coupling between the DS theta frequency (top-right) and HPC high-gamma or between the HPC theta frequency and DS high-gamma (bottom-right).

## Discussion

**Figure 6:**
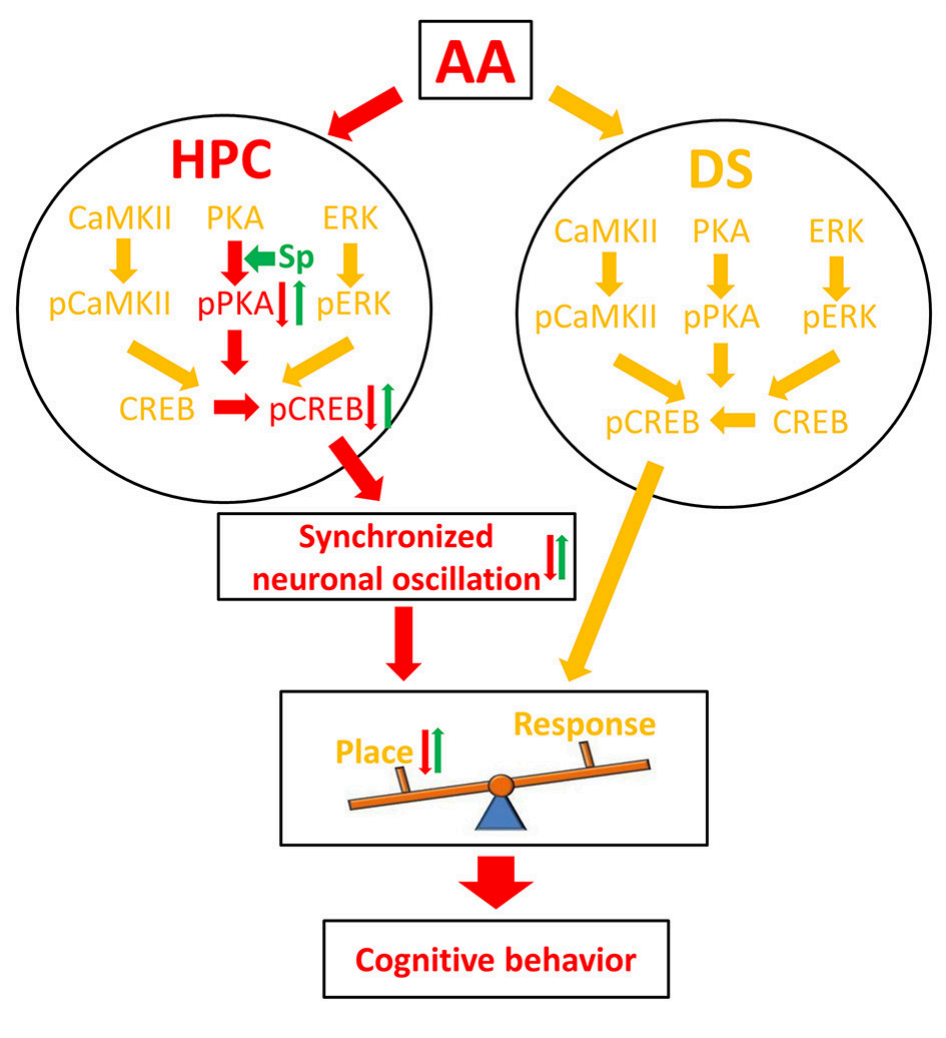
Schematic illustration of a molecular mechanism for AA effect on learning strategy. Red arrows denote the pathway under the inhibitory effect of AA; green arrows denote the reversal effect of Sp-cAMP; yellow arrows denote the pathways that did not affect by AA. AA, acute alcohol; HPC, the dorsal CA1 region of the hippocampus; DS, the dorsal striatum; Sp, Sp-cAMP. Place, place strategy; Response, response strategy.

The HPC and the DS are conventionally viewed as cooperative (Kim and Baxter, 2001; Voermans et al., 2004) or competitive (Kim and Baxter, 2001; Poldrack et al., 2001) learning and memory systems. Our behavioral experiment demonstrates that AA exposure before the learning session attenuates the use of the HPC-dependent spatial strategy and causes a switch to a response strategy that relies on the DS to locate the food reward in the T-maze task. To explore the underlying mechanism, we analyzed independent and coordinated oscillation between the two brain regions and found a series of novel results (Figure 6). First, the training-induced increase in pCREB was reduced in AA-treated rats' HPC, but not DS. The finding was thought to underlie the preferred response-strategy behavior. AA disrupted the PKA/CREB, but not the CaMKII/CREB or pERK/CREB, pathway via inhibition of pPKA activity. Second, the intra- and cross-structure couplings were strengthened in rats that applied a place strategy, but depressed in rats that applied a response strategy. As expected, the reductions of synchronized neuronal oscillations contributed to the change in response-strategy of AA-treated rats. Third, the infusion of the PKA activator could mitigate the response-strategy bias and spatial deficits, and it strengthened functional couplings. Our findings suggest that the brain compensates for negative effects of the AA on the HPC memory system by using the DS memory system.

The shift in learning strategy found after low-dose AA treatment (Figure S2), which did not affect the performance on a similar Y-maze apparatus or in an open-field task, eliminated the possibility that the strategy preference was due to AA-induced defects of locomotion and motivation. However, high-dose AA affected both exploratory and locomotor function, which was in line with previous reports (Chappell and Weiner, 2008; Blednov et al., 2014). Since this effect probable caused spurious consequences on behavioral tests (Gould, 2003), the findings in T-maze could not be evidenced they visited arms using cognitive strategy. Intriguingly, in a non-spatial dependent test, rats receiving low-dose ethanol (1.0 g/kg) successfully showed recognition behavior, while rats receiving higher dose of ethanol (3.0 g/kg) could not complete the test (Spinetta et al., 2008). Therefore, these findings combined with ours implied that the impact of alcohol exposure on cognitive behaviors differed in dose or BAC (Chandra et al., 2008; Van Skike et al., 2012). Moreover, we found both doses induced spatial defects in classical MWM task, which was consistent with previous reports (Givens, 1995; Matthews et al., 1999). Obviously, there were conditions and tasks for which no alternative strategies were available. Interestingly, post-training alcohol enhanced reactivated fear memories in a contextual fear conditioning task (Nomura and Matsuki, 2008) and improved the performance in a short-term social memory task (Prediger and Takahashi, 2003), but impaired memory retention of appetite-motivated discrimination (Land and Spear, 2004). Thus, the availability of alternative learning strategies and mechanisms that can compensate for an HPC/DS dysfunction may determine whether or not AA has noticeable effects on cognitive performance. This shift from an HPC-dependent to a DS-dependent learning strategy under AA conditions may also explain some of the discrepancies in the literature (Weitemier and Ryabinin, 2003; Gulick and Gould, 2007).

Interference with the HPC CREB-mediated transcription reportedly impaired spatial learning and facilitates response learning (Martel et al., 2006; Lee et al., 2008). Moreover, elevated DS CREB expression impaired memory for place training (Kathirvelu and Colombo, 2013). These findings demonstrate that the competitive interactions between discrete neural systems are mediated by CREB function. Our findings suggest that the preferred-strategy effects of AA may result from reduced HPC, but not DS, pCREB expression. Previously, AA was found to reduce hippocampal and cerebral pCREB levels in an age- and brain-region–specific manner (Chandler and Sutton, 2005). Interestingly, AA caused an HPC pCREB reduction during treatment but an increase during subsequent withdrawal (Bison and Crews, 2003). Our current results are also consistent with the report that glucose-induced competition between neural systems may involve pCREB (Morris et al., 2013). Moreover, a study revealed a suppressive effect of alcohol on CREB activity in a dose-dependent manner, while total CREB levels remained unaltered upon alcohol treatment (Sakai et al., 2005). Therefore, both previoius and the current findings verified that the decline in pCREB levels was not due to the reduction of total CREB levels. Furthermore, our findings clearly showed that the inhibitory effect of AA on pCREB, but not CREB, was attributed to the performance of obstinately using the response-learning strategy. Reports of learning-induced pCREB have indicated that the time courses of peak phosphorylation may vary (Colombo et al., 2003; Zhang et al., 2003). For example, rats that adopted a response strategy were found to have elevated DS pCREB levels immediately after learning (Colombo et al., 2003). After 1 h of learning, the sustained high-level expression of pCREB in the HPC was observed in place but not response-strategy animals (Colombo et al., 2003). Our finding extends previous reports that differences in the number of training trials could cause differences in the expression of pCREB/pPKA. However, we found that AA reduced PKA-dependent, but not ERK or CaMKII-dependent, CREB phosphorylation (Figure S10). Inhibition of activity-dependent PKA activation with AA exposure resulted in a similar inhibition of activity-dependent CREB activation, which induced behavioral deficits. For example, PKA regulatory RIIβ subunit mutant mice, which exhibited lower total cAMP-stimulated PKA activity, were less sensitive to the sedative-hypnotic effects of alcohol (Lai et al., 2007). However, in primary neuronal cultures alcohol inhibition of ERK activity was exactly mirrored by changes in CREB activity (Chandler, 2003). Another study showed that AA (3.5 g/kg) reduced pERK levels in the cerebral cortex during the brain growth-spurt period (Chandler and Sutton, 2005). Although it is clear that PKA also plays a role in AA-mediated behavior, the processes are complex and may change when experimental factors are varied.

The variability of oscillations suggests that both the HPC and the DS are dynamic and highly task-dependent rather than having merely a single temporal relation (DeCoteau et al., 2007; Tort et al., 2008; Thorn et al., 2010). Our current findings confirm that the HPC and DS LFP oscillations might themselves be interrelated as animals learn and perform sequences of actions. Compared with the HPC, the DS was found to generate more stereotypical and less flexible responses, and rats experienced more difficulty in adapting to changing conditions (Hartley et al., 2003; Hartley and Burgess, 2005). Regardless of learning behaviors, the DS continuously integrates relevant information that is needed to obtain rewards (Regier et al., 2015). Similarly, location-specific neural activity by the DS neurons was observed during both place and response task performance after a context change (Yeshenko et al., 2004). This interpretation of the functional significance of striatal processing is consistent with our finding of the powerful DS phase-locking between spikes and high-theta oscillation in both learning strategies. An intact striatum is necessary to correctly use cognitive strategies (Monchi et al., 2006; Thorn et al., 2010). However, it may not be sufficient for processing the place-related strategy, since we found that the functional coupling between the HPC and DS was strengthened during the place-learning strategy. The cross-structure couplings were highly frequency-dependent, suggesting that using the place-strategy relies on the specific coupling of the HPC-DS rhythms rather than simply a general synchronization of the LFP activity. Multiple co-existing patterns of phase coupling occur and might actually better characterize different learning states (Tort et al., 2008, 2013; van Wingerden et al., 2014). Moreover, in the intra-structure of both brain regions, single spikes relative to the theta phase that coupled cross-structure would gate or convey information during cognitive behavior (Lisman, 2005; Tort et al., 2008). More importantly, the failing to find the activity ratio difference between two regions indicates the selective effect is not associated with behavior strategy (Figure S5E). Our observation of the cross-structure HPC-DS coupling strikingly demonstrated that two regions could interact at multiple levels during strategy-switch manipulation, and the details of their responses differed (Eschenko and Mizumori, 2007), which was consistent with the postulated differences between the HPC and DS contributions to learning and memory systems.

Although the suppressive effects of AA on the HPC theta (6–9 Hz) phase induced spatial working memory deficits (Givens, 1995), our results show that this effect is not directly related to learning strategy deficits (Figure S7). The rigid striatal-dependent performance was the most direct explanation for the reduction in memory flexibility after AA treatment (Wright et al., 2013; Abramson et al., 2015), so it would be of interest to determine if AA also affects the neural activities of other regions, such as that involved in reversal learning (Ragozzino, 2007). The decrease in cross-structure couplings could be thought of as an inhibitory effect of AA on synchronized oscillations (Lee and Yun, 2014) and functional connectivity in cortical activity (Levin et al., 1998; Meda et al., 2009). The high-frequency coupling has found to be involved in different implicit learning tasks (Wessel et al., 2012). Particularly, the depressed fast frequency bands are also well documented characteristics of AA exposure (Wang et al., 2016), supporting the suppressive effect of AA on amplitude-phase couplings. More importantly, the changing patterns of phase coherence (DeCoteau et al., 2007; van Wingerden et al., 2014) and the intra-structure phase-locked units (Csicsvari et al., 2003; Fries, 2005) are the cardinal features of the neural activity that accompanies the learning strategy (Figures S8, S9). Therefore, our findings suggest that the defective functional couplings, which are much weaker in the response behaviors, reflect the effect of AA on the preferred strategy.

Behavioral pharmacological results clearly showed that the selective effect of Sp-cAMP on the memory acquisition resulted in attenuation of the AA-induced response-strategy bias (Figure S6). In line with our observations, systemic administration of a high dose of Sp-cAMP (2.1 nmol) was found to induce locomotor impairment (Ramos et al., 2006; Paine et al., 2009). Most importantly, regulation of the HPC-specific cAMP-PKA-CREB signaling cascade by Sp-cAMP could strengthen the intra- and cross-structure synchronized neuronal oscillations and mitigate the AA-induced response-strategy bias. Extensive evidence indicates that AA-induced inhibition of neural oscillations not only causes strategy bias but also selectively impairs spatial learning (White et al., 2000; Ketchum et al., 2016) and working memory (Givens, 1995; Givens et al., 1998). The findings of behavioral studies were consistent with this view and demonstrated that this selective impairment cannot be attributed to the alcohol dose (Matthews and Silvers, 2004) or inability to visualize distant information (White et al., 1998). From the 12-trial location learning test, we found AA-treated rats used the sustained patterns of non-spatial strategy rather than spatial strategy, which could also be interpreted as reflecting learning flexibility deficits (Abramson et al., 2015). Similarly, alcohol was found to impair the performance by interfering with their ability to acquire and use required water maze behavioral strategies (Cain et al., 2002). This improper strategy selection was rescued by Sp-cAMP treatment, indicating that these changes were primarily responsible for the impaired spatial ability. Moreover, previous studies have also implied the significant role of the PKA/CREB pathway in the extracellular field potentials (Sanyal et al., 2002; Waltereit and Weller, 2003; Barco and Marie, 2011). Therefore, our findings extend the understanding of the effects of AA on spatial learning and cognitive flexibility, which are mostly attributed to the disruptive effect of AA on the use of the place-strategy. Further evidence may come from transgenic animals and optogenetics.

In conclusion, our study indicates that the brain could temporarily compensate for the effects of AA on cognitive performance by switching to alternative learning mechanisms. An important implication of these findings is that the effects of AA in the brain are always evident on spatial, but not non-spatial, learning, and cognitive flexibility. More importantly, the modification of pCREB expression by a PKA activator could reverse the inhibitory effects of AA on intra- and cross-structure synchronized neuronal oscillations, which is attributed to the response-strategy bias. The reversal effect indicates a potential new avenue toward treatment of spatial learning deficits associated with AA in the future.

## Ethics Statement

All procedures were done in accordance with ethical guidelines laid down by the ethics Committee on the Care and Use of Animals Committee of Guangzhou University of Chinese Medicine.

## Author Contributions

LA, WS, and CT conceived and designed the experiments. LA, WS, and XL performed the experiments and analyzed the data. LA and WS wrote the manuscript.

### Conflict of Interest Statement

The authors declare that the research was conducted in the absence of any commercial or financial relationships that could be construed as a potential conflict of interest.

## Abbreviations

AA: acute alcohol
BAC: blood alcohol concentration
CaMKII: calmodulin-dependent protein kinase II
camp: adenosine 3′: 5′cyclic monophosphate
CREB: cAMP response-element binding protein
DS: dorsal striatum
ERK: extracellular signal-regulated kinase
FSI: fast spiking interneuron
HPC: dorsal CA1 region of the hippocampus
MI: modulation index
MSN: medium spiny neuron
MWM: Morris water maze
NMDA: N-methyl-D-aspartate
pCaMKII: phosphorylated calmodulin-dependent protein kinase II
pCREB: phosphorylation of cAMP response-element binding protein
pERK: phosphorylated extracellular signal-regulated kinase
PKA: cAMP-dependent protein kinase
pPKA: phosphorylated cAMP-dependent protein kinase
Sp-camp: Sp-Adenosine 3′, 5′-cyclic monophosphorothioate triethylammonium salt hydrate
TAN: tonically active neuron.
