# Supplementary material for: Acute Low Alcohol Disrupts Hippocampus-Striatum Neural Correlate of Learning Strategy by Inhibition of PKA/CREB Pathway in Rats
Source: Front Pharmacol. 2018 Dec 6;9:1439. doi: 10.3389/fphar.2018.01439 (PMC6291496; doi:10.3389/fphar.2018.01439)
Supplement: Supplementary file 1 [file Presentation_1.pdf]

Supplementary information

SI Materials and Methods.

Unit isolation and classification criteria.

Spike sorting was performed with offline Neuralynx's software (SpikeSort 3D), using a combination of KlustaKwik, followed by manual adjustment of the clusters (Klusters software package). Briefly, multiple parameters were used to effectively visualize clusters with the most often used combination of spike height, trough, and energy, associated with the waveforms. Each cluster was then checked manually to ensure that the cluster boundaries were well separated and waveform shapes were consistent with action potentials (Fig. S4A). Interspike interval histograms were additionally examined for ensuring single unit activity (inserts of Fig. S4). Using methods described elsewhere (1), units were then graded for quality and classified as putative medium spiny neuron (MSN), fast spiking interneuron (FSI), or tonically active neuron (TAN) subtypes form striatal recording and pyramidal neuron and putative interneuron from hippocampal recording, respectively. A pyramidal neuron or MSN was further classified as "related-to-task" if its firing rate in any ±300 ms perievent window was more than 2 standard deviations (SD) above its baseline firing rate for three consecutive 20 ms bins (2). Units not classified as task-responsive were deemed "unrelated-to-task."

Neural activity in behavioral task

Behavioral correlates of neural activity changes were assessed by constructing perievent time histograms synchronized during task-related event periods (i.e., 2 sec before trial start as baseline, 0.5 sec before to 1.0 sec after trial start, 0.5 sec before to 1.0 sec after turn start, 0.5 sec before to 1.5 sec after trial end, 1.0 sec after rewarding). Firing rates in 500-ms bins were each compared against the firing rates from the basal recording (from 90 sec pre-trial initiation to 30 sec pre-trial initiation). Significance was tested by using ANOVA analyses (P<0.05). The neuron was related to the

occurrence of the event only if one test bin from it significantly differed from all baseline bins (3). Neurons were classified into four categories based on the task period in which they exhibited significant firing-rate changes (Fig. S4A and S5A; for HPC: change in relation to turn (start to end), movement in the aim arm or reward; for DS: change in related to trial start, turn start or reward). The time windows of task event were based on visual inspection of the data. Similarly, z-score (subtracting firing rate averages and dividing by the SD) was compared to ensure that a minority of neurons with exceptionally high-firing rates did not dominate the average.

To compare the alternation of neural activity between HPC and DS following alcohol exposure, the distribution of the ratio of HPC activity to DS activity was calculated. Briefly, total number of cells and total number of turn-related cells from each recording day were collected and grouped by strategy that rats used. Percentage of each brain area activity was calculated as turn-related cell number divided by total cell number. Then the characteristic of neural activity between HPC and DS when the strategy was applied were obtained (Fig. S5E). The ratio of HPC activity to DS activity was calculated as the percentage of HPC activity divided by the percentage of DS activity. The distribution was determined by the learning strategy obtained from behavioral task under alcohol or saline condition. Each single data was collected by each recording day, thus the mean and S.E.M. was obtained through the whole recording days (Fig. S5E insert).

Spectral analysis of LFPs

Spectral analysis was used to assess the dominant frequencies in the LFPs during the task-related event periods (as above). The analysis of LFP power was performed by using Neuroexplorer (Nex Technologies, Littleton, MA). The power spectra were calculated using Welch's method (1024 frequencies between 1 and 200 Hz, smoothed with a Gaussian Kernel with bin width 3). Then the low-theta (4-7 Hz), high-theta (7-12 Hz), low-gamma (30-48 Hz), high-gamma (52-120 Hz) and high-frequency (120-200 Hz) were obtained by band-pass filtering. The mean power spectral density (PSD) in each given band was calculated.

SI Results.

1. Behavioral Section.

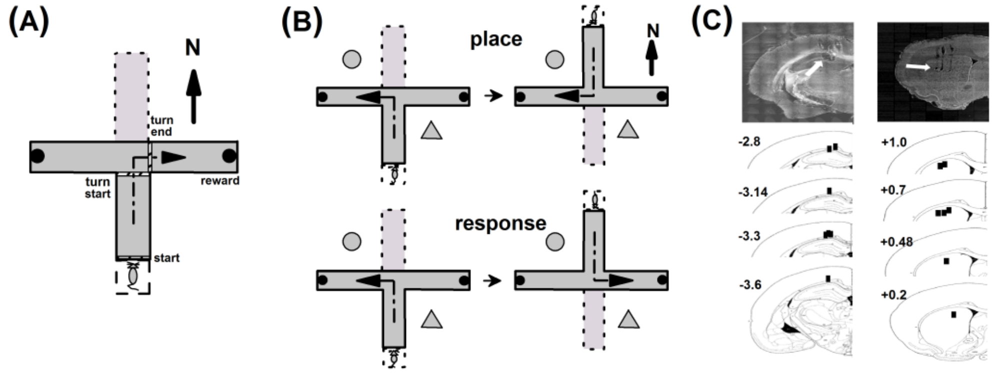

Fig. S1. Schematic and histological verification. (A) T-maze with task events. Gary bars indicate event initiated when rats' hind-paws crossed them. **(B)** Schematic illustrations of place strategy (top) and response strategy (bottom). **(C)** Histological verification of recording sites in the HPC (top-left) and DS (top-right). The white arrows represented the tetrode sites. Graphs adapted from Rat Brain Atlas of Paxinos and Watson (1997). Tetrode tracks in the HPC (bottom-left) and DS (bottom-right) were represented by solid black rectangles.

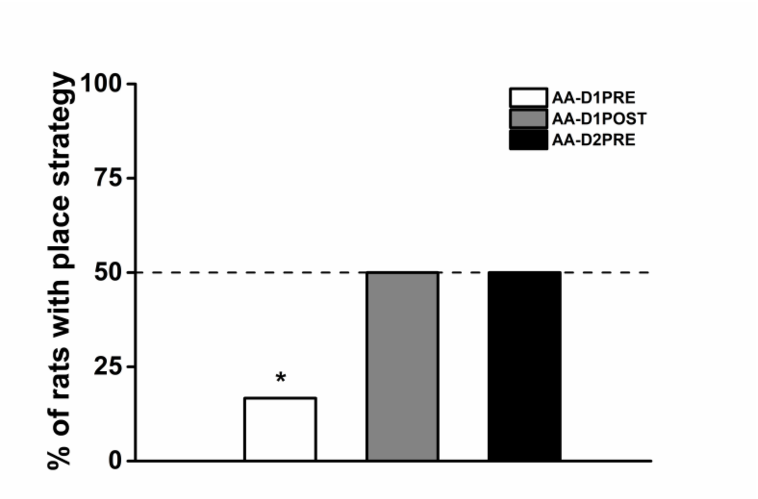

Fig. S2. The effect of AA on using learning strategy immediately after a three-session training or 30 min before probe test. The percentage of animals that used a place strategy was calculated and presented. Rats received a probe trial starting

from the opposite start arm. Compared with AA-D1PRE group (treated 30 min before training), a higher number of rats with place strategy was found when AA was treated immediately after training (AA-D1POST; Chi-square test: χ^2=5.63, df=1; P<0.05) or 30 min before probe test on the 2nd day (AA-D2PRE; Chi-square test: χ^2=5.63, df=1; P<0.05). *P<0.05 versus only AA-D1PRE. Data are shown as mean ± SEM. n=6 for each group.

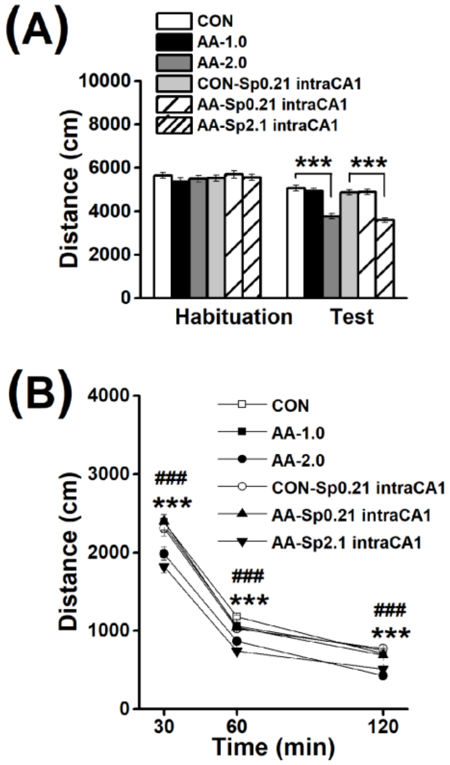

Fig. S3. Effect of AA (1.0 or 2.10 g/kg) and Sp-cAMP (0.21 or 2.1 nmol) on locomotor activity. To exclude the specific effect of Sp-cAMP on spontaneous behavior, we assessed on locomotor activity in an open field. The total distances (top) during the habituation and test session. (***P<0.001). During habituation day, no difference was found in total locomotor activity. The time courses of activity during the test session (bottom). ANOVAs yielded a significant between-groups difference in time-course results ($F_{(5,\ 30)}$=13.03, P<0.001). Locomotor activity was significantly lower in AA-Sp2.1-intraCA1 group compared with AA-Sp0.21-intraCA1 and CON-Sp0.21-intraCA1 groups at each time point (P<0.001 for all comparisons). These results rule out the possible that locomotor effect by low-dose Sp-cAMP

impacted the behavioral strategy improvement. High-dose of alcohol also reduced traveled distance on each session compared to control and AA-1.0 group (P<0.001 for all comparisons). These findings confirmed the high, but not low, dose of alcohol, disrupted locomotor behavior. (***P<0.001, AA-2.0 vs. CON or AA-1.0; ###P<0.001, AA-Sp2.1-intraCA1 vs. CON or AA-Sp0.21-intraCA1). n=6 for each group.

2. Electrophysiology Section

2.1 Differential effects of alcohol on firing patterns and proportion in the HPC and DS

Neuron spike trains were electrophysiologically recorded and classified by waveform shape and spiking patterns (see SI Materials and Methods for explanation). Totally, 734 single neurons were isolated from the HPC and DS of 16 rats. As shown in Fig. S4, wide-waveform neurons were classified as hippocampal pyramidal neurons (143 from control group, 85.1% of the population; 132 from AA group, , 82.0% of the population) and striatal MSN (152 from control group, 75.6% of the population; 161 from AA group, 78.9% of the population) and TAN (7 from control group, 3.5% of the population; 8 from AA group, 3.9% of the population), while narrow-waveform neurons were classified as hippocampal putative interneurons (13 from control group, 7.7% of the population; 15 from AA group, 9.3% of the population) and striatal FSI (17 from control group, 8.5% of the population; 16 from AA group, 7.8% of the population). Additionally, twenty-six cells can not classified from HPC (12 from control group, 7.1% of the population; 14 from AA group, 8.7% of the population), and forty-four cells can not classified from DS (25 from control group, 12.4% of the population; 19 from AA group, 9.3% of the population). Histological procedures confirmed electrode placement for both HPC and DS electrodes (Fig. S1C). Examination of the recording tracks confirmed that HPC units were recorded both in CA1 region of dorsal hippocampus. Striatal units were confined to dorsal-medial and

dorsal-lateral striatum. Data were combined across areas since we did not detect regional specificity of the responses. The fact that many more striatal neurons were recorded in DS than HPC was likely due to the failure of a few electrodes on HPC drives, and did not reflect a meaningful difference in information representation.

To understand the effect of alcohol on neural response to strategy behavior, we then sought to examine how the firing pattern of individual neurons changed in HPC and DS regions. Since the rats was well-trained before recording (average 615 trials), the neural firing patterns of HPC (Fig. S5A left) and DS (Fig. S5A right) were very familiar during the T-maze performance. Generally, three main categories of location-specific firing fields could be distinguished from the HPC of control group. The first group of cells showed activity at the center of the T-maze (turn-decision related); firing fields mainly covered the center area, sometimes including the transition areas between the platform and the three arms (33.6%; turn-related; Fig. S5A top-left). Another group exhibited firing fields covering most of the selected arm (14.0%; aim arm related), and did not show firing patterns when rat was placed in the other un-chosen arm (data not shown). The third group showed the firing fields located near specific reward bowl (11.2%; reward-related). By comparison, the proportions of these three typical cells were reduced drastically in alcohol group (Fig. S5A top-right). The ratios of turn-related, aim-related and reward-related cells were 15.2%, 16.2%, and 11.2%, respectively. DS units showed spatial firing fields that generally covered large areas of the T-maze (Fig. S5A bottom-left). None of pyramidal neurons displayed firing fields at similar locations in each of the chambers, despite the fact that the chambers were identical. Above one third of the cells emitted spikes in these central portions (38.2%; turn-related). Another substantial portion of cells fired at the start place (11.4%; start-related). A last subgroup comprised cells fired specifically at, or near reward blow (11.4%; reward-related). However, the proportions of turn-related (39.2%), start-related (14.0%) and reward-related (15.2%) cells in alcohol group were not obviously different from control group (Fig. S5A bottom-right). MSN cells exhibited multiple firing fields that were combinations of the three main groups.

The neural activities were collected and analyzed during the event periods as illustrated in Fig. S1A. The turn, but not reward or aim arm, related HPC pyramidal neurons recorded in the alcohol-treated group fired at markedly lower rate than those in the control one ($P<0.001$; Fig. S5B left). Similar results were also found in z-score analysis during turn-decision period ($P<0.01$; Fig. S5C left). However, no difference was observed in the spike frequency or z-score during the baseline or trial start period between two groups, although firing rate at turn, aim arm and reward moments were higher than their baseline ($P<0.001$, for all comparisons). The striatal MSNs had higher firing rates in alcohol group during start, turn and reward periods compared with its baseline ($P<0.0001$, for all comparisons; Fig. S5B right). Higher firing rates were also found during event periods, including start ($P<0.001$), turn-decision ($P<0.001$), and reward ($P<0.001$) in control group. However, their activity was equally enhanced in both groups. Normalized firing rates by z-score analysis also found firing frequency of control group and alcohol group elevated during events ($P<0.001$, for all comparisons; Fig. S5C right). Consistent with previous reports, we found the selective inhibitory effect of alcohol on HPC, but not DS, cerebellum, lateral septal nucleus or prefrontal cortex (4, 5). The firing rate and its normalized z-score in each strategy have also been considered within each condition (control or alcohol). However, no statistical difference was found between the strategies (data not shown). Since the magnitude of the neuronal activity did not depend on the use of place or response behavioral strategy, we combined the results of two strategy groups in each condition.

The distribution of the turn-related pyramidal neurons in alcohol group was statistical different (Chi-square test: $X^2=4.69$, df=2; $P<0.05$, Fig. S5D left), which was tended to decrease. There is no change in the proportion of other task-related cells or cells were not related to task events. However, the population of turn-related MSNs did not appear differently (Chi-square test: $X^2=0.07$, df=2; $P>0.05$, Fig. S5D right). The characteristic of activity pattern between HPC and DS was shown in Fig. S5E. There was no significant correlation between strategy and HPC activity (Pearson's r = 0.021, $P>0.05$), between strategy and HPC activity (Pearson's r = 0.016,

P>0.05), or between strategy and the ratio of HPC activity to DS activity (Pearson's r = 0.08, *P*>0.05). The activity ratio of HPC to DS in CON groups was higher than in AA groups (*P*<0.001, for all comparisons; Fig. S5E insert).

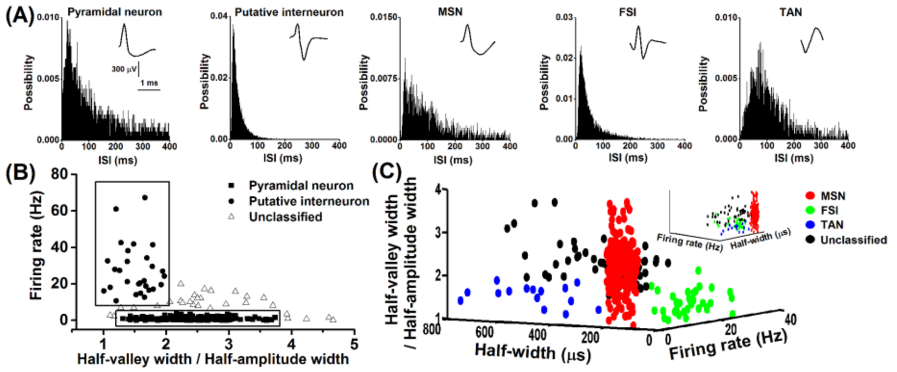

Fig. S4. Unit classification. (A) Interspike interval (ISI) histogram and an average spike waveform (inserts) were shown for hippocampal pyramidal neuron and putative interneuron, and striatal medium spiny neuron (MSN), fast spiking interneuron (FSI), or tonically active neuron (TAN). **(B)** Distribution of average firing rate and half-valley to half-peak ratio of each hippocampal neurons. The peak–valley ratio correlated positively with the spike width (Pearson’s *r*=0.37; *P*<0.001). Therefore, the wide spikes tended to have large half-valley to half-peak ratio. On the other side, a significant negative correlation between the mean firing rate and the ratio of half-valley to half-peak of the spike waveform was found (Pearson’s *r*=–0.26; *P*<0.001). So the high firing rate cells tended to have spike waveforms with relatively small half-valley to half-peak ratio. Cells from hippocampal area were therefore separated into two groups. **(C)** Distribution of average firing rate, half-width of waveform and half-valley to half-amplitude ratio of each striatal neurons. The insert was obtained from another angle of view. The three populations of striatal neurons displayed distinctive firing patterns. A significant negative correlation between the mean firing rate and the ratio of half-valley to half-peak of the spike waveform (Pearson’s *r*=–0.33; *P*<0.001) so that high frequency of FSI tended to have spike waveforms with relatively small half-valley to half-peak ratio. Both MSN and TAN had broad waveform, while the frequency of TAN was higher than MSN

(*P*<0.001). MSN had the largest the ratio of half-valley to half-peak, with statistical difference compared with FSI (*P*<0.001) and TAN (*P*<0.001). The neuron number of each group was indicated in the 2.1 section.

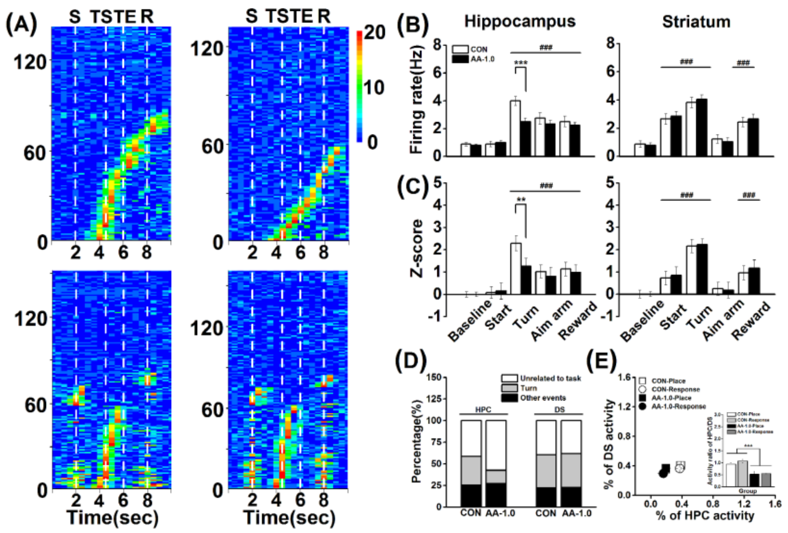

Fig. S5. Spiking activity in rat HPC and DS during strategy training. (A) Selective disruption of activity of HPC projection neurons by AA. Ensemble activity of pyramidal neurons recorded in the control (top-left) and AA-impaired (top-right) HPC, and MSNs recorded in the control (bottom-left) and AA-impaired (bottom-right) DS. Firing rate of each unit during the whole task of 4 events (as labeled on the top, S indicated trial start; TS indicated turn start; TE indicated turn end; R indicated rewarding). Color scale was shown at right. Numbers of units for the stage were indicated to the right of each row. **A**verage baseline, trial start, turn (turn start to turn end), aim arm (turn end to rewarding) and rewarding firing rates **(B)** and z-score **(C)** of HPC pyramidal neurons (left), and DS (right) MSNs. (***P*<0.01; ### or ****P*<0.0001). Hash-tag indicated significant difference compared with their matched baseline. **(D)** The percentage of cells engaged in task events: turn, other events except turn, and unrelated to task. Data from HPC was shown on the left, DS was shown on the right. **(E)** The distribution of DS activity and HPC activity in difference strategy condition. Insert: the average neural firing ratio of HPC to DS. (****P*<0.0001). The neuron number of each group was indicated in the 2.1 section.

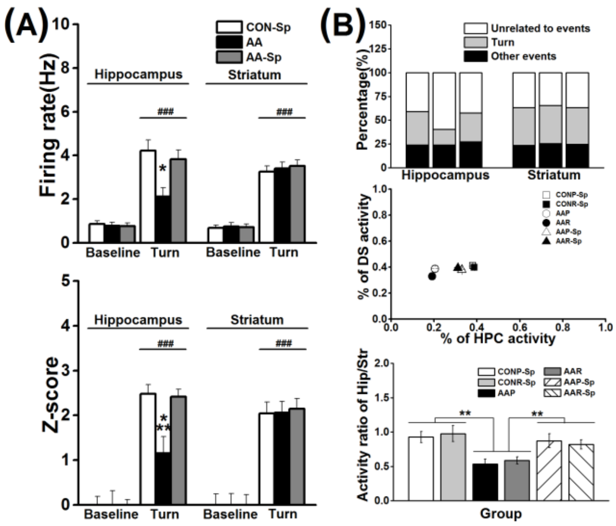

Fig. S6. Effect of Sp-cAMP on HPC and DS neural activiies of AA-treated rats during decision-making period. (A) The firing rates (top) and z-score (bottom) during average baseline and turn-decision period of HPC pyramidal neurons (left), and DS MSNs (right). (**P*<0.05,****P*<0.001, vs. CON-Sp or AA-Sp; ###*P*<0.001, vs. matched baseline). **(B)** The percentage of cells involved in turn-decision, other events except turn-decision, or unrelated to task (top), the distribution of DS activity and HPC activity in strategy learning (middle), the neural firing ratio of HPC to DS (bottom). (***P*<0.01). *n*=7 for each group. Infusion of Sp-cAMP effectively enhanced the HPC turn-related firing rate (*P*<0.001), as well as z-score (*P*<0.001). The percentage of cells that fired during turn period was also increased, without alteration of other event-cell proportion. No statistical difference in firing rate, z-score, percentage of turn-decision cells, or the activity pattern between HPC and DS was observed in the DS after injecting Sp-cAMP to alcohol rats. Noticeably, the elevated activity ratio of HPC to DS (*P*<0.01) indicated that the functional improvement in the HPC after Sp-cAMP treatment. The neuron number of each group was indicated in the 2.1 section.

2.2 Effect of alcohol on power oscillatory activity of HPC and DS during strategy task.

We recorded LFPs simultaneously in the HPC and DS as rats performed the session III of learning period (Fig. S7A and Fig. S7B). Both HPC and DS low-theta (4-7 Hz)

remained nearly constant. The power of both HPC and DS low-gamma (30–48 Hz) declined abruptly as the rats left the start chamber, but gradually rose as the reward zone was approached. By contrast, the high-frequency (120-200 Hz) oscillation of HPC and DS reached to peak near the turn area and then declined to baseline level slowly. ANOVA analysis indicated main effects of event on low-gamma ($F_{(4, 112)}$=3.87, P<0.01) and HF ($F_{(4, 112)}$=4.80, P<0.01), without effects of treatment. For high-theta (7-12 Hz), HPC oscillation peaked toward the turn-decision period, but diminished gradually, while a sharp peak occurred in the DS around the turn-decision period and then fell back to basal level. ANOVA analysis revealed a main effect of treatment on HPC high-theta oscillation ($F_{(1, 30)}$=85.83, P<0.001), with lower power observed during the baseline and task periods compared alcohol to control groups (baseline, turn, and aim arm P<0.001; start, P<0.05; reward, P<0.01). Intriguingly, these differences were not found when the power was normalized by baseline (Fig. S7A insert of the second image), indicated the functional alteration of power amplitude between alcohol and control groups was similar. A main effect of strategy on DS high-theta oscillation was found ($F_{(1, 30)}$=15.79, P<0.001). Tukey's test found a higher power in response strategy groups than in place ones at turn-decision period (P<0.001, CON-R vs. CON-P; P<0.001, AA-R vs. AA-P). Similar results were found when the power was normalized ($F_{(1, 30)}$=8.09, P<0.01; Fig. S7B insert of the second image). The power of HPC high-gamma (52-120 Hz) band was rapidly increased at turn-decision area and kept slight increasing later, while DS high-gamma power reached a peak near turn-decision period and then fell. ANOVA analysis failed to detect a main effect of treatment or strategy on DS high-gamma power. Nevertheless, a main effect of treatment was found in HPC ($F_{(1, 30)}$=104.71, P<0.01), with a statistical increasing compared control with alcohol group (baseline and start, P<0.01; turn, aim arm and reward, P<0.001). There was no difference in normalized power in HPC region (Fig. S7B insert of the fourth image). Overall, although alcohol inhibited HPC, but not DS, theta and gamma power during behavioral task, while functional HPC power of alcohol-treated rats kept a similar neural correlate with behavioral events as controls. The strategy-specific increasing power at DS high-theta band

suggested the essential role of DS in response strategy task, particularly at turn-decision period.

These findings indicated that the suppressive effect of AA treatment on high-theta and high-gamma oscillations from HPC should not be attributed to the strategy bias. Moreover, DS high-theta power was not affected in AA-treated rats that used response strategy.

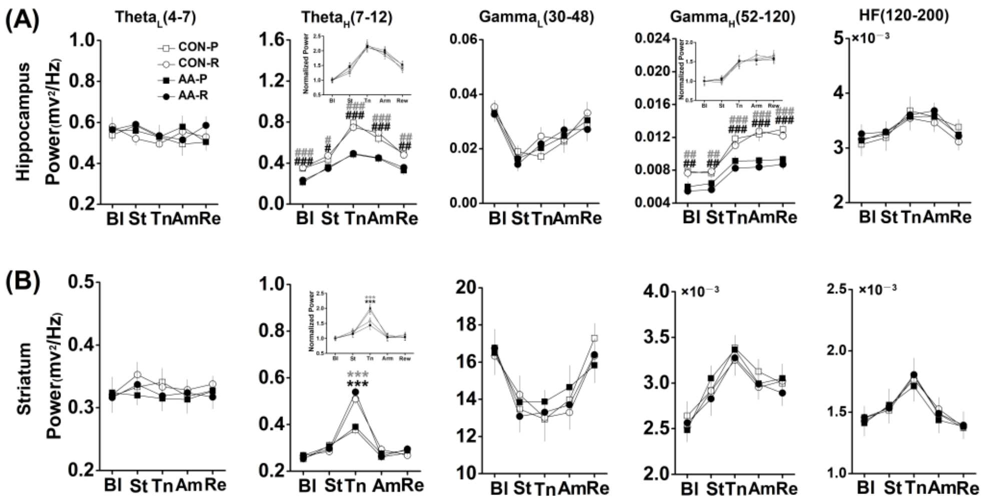

Fig. S7. Effects of AA on local field potentials during strategy learning. (A) Average power of HPC oscillations for successive event periods over 5 frequency ranges. **(B)** Average power of DS oscillations for successive event periods over 5 frequency ranges. The inserts were the normalized power by each baseline (if applicable). Event labels: Bl, baseline; St, start; Tn, turn start to end; Am, choice arm period; Re, reward. (#P<0.05, ##P<0.01, ###P<0.001; black hash: CON-P vs. AA-P; gray hash: CON-R vs. AA-R.***P<0.001; black asterisks: CON-P vs. CON-R; gray asterisks: AA-P vs. AA-R). n=7 for each group.

2.3 Effect of alcohol on synchronized neuronal oscillations during strategy-learning task.

Since both LFP and neural activity were related to learning task while no strategy-specific alternation was observed in these analyses (Fig. S5), we therefore examined the relationship between the firing of individual neurons and the

simultaneously recorded LFPs in each brain area during the turn-decision period (Fig. S8A). As shown in Fig. S8B, the mean vector length were in a similar level in low-theta, low-gamma, and high-gamma of HPC and DS. Significant effects of strategy ($F_{(1, 30)}$=41.89, P<0.001) and treatment ($F_{(1, 30)}$=67.02, P<0.001) on high-theta band was found. Tukey's test revealed there was a significant enhanced phase-locking compared place-strategy to response-strategy (P<0.001, CON-R vs. CON-P; P<0.001, AA-R vs. AA-P). The vector length of AA-treated group (AA-R), which tend to apply response strategy, was even lower than control response-strategy group (CON-R; P<0.05). The vector length AA-treated place-strategy group (AA-P) was also smaller than control place-strategy group (CON-P; P<0.01). The mean phase of HPC neurons tended to fire at the peak of the high-theta cycle in CON-P, CON-R and AA-P groups, but not in AA-R group (Fig. S8C left). These results indicated a powerful phase-locking of HPC high-theta oscillation was essential for a place strategy but weakened in response-strategy AA condition. Interestingly, the prominent firing phase advanced alcohol-treated animal to apply a place but not response strategy. DS neurons also had an unimodal population distribution, which also occurred at the peak (Fig. S8C right). Therefore, the phase-locking of DS high-theta oscillation either was not essential for a response-strategy or played a critical role for both place and response strategies.

Since DS and HPC were considered to be functionally related (6), the phase-amplitude coupling between two regions were calculated (Fig. S9A). Analysis of the mean peak coherence values between the HPC and DS revealed a significant main effect of strategy across high-theta frequency during the tuning period ($F_{(1, 30)}$=56.60, P<0.001). However, no effect of response strategy was observed within the other bands. Tukey's test revealed that the coherence of CON-P was higher than CON-R (P<0.001). Similar result was also found between AA-P and AA-R (P<0.001). No main effect on treatment was observed across frequency bands investigated. These results indicated place-strategy was associated with a higher coherence between the HPC and DS, while response-strategy was associated with a lower coherence.

Since the preferred low-frequency rhythm modulating high-gamma amplitude was

dependent on the task-specific modality (7), we tested whether the phase-amplitude coupling was associated with the strategy selection. The phase-amplitude couplings between the DS high-theta phase and the amplitude of HPC fast-frequency oscillations were very prominent in place strategy groups (Fig. S9A, first-row, first-image and third-image). However, this effect was much weaker in response strategy groups (Fig. S9A, first-row, second-image and fourth-image). ANOVA analysis indicated a main effect of strategy (Fig. S9B middle), but no effect of treatment on the mean peak of modulation index (MI). This different did not resulted from specific band effects, since the statistical differences were identified in two common selected bands, 120-160 Hz ($F_{(1, 30)}$=83.17, P<0.001) (8) and 120-200 Hz ($F_{(1, 30)}$=94.05, P<0.001) (6). Although the DS theta modulation of HPC 55-120 Hz (high-gamma) oscillations was found (Fig. S9B, first-row), there was no statistical different among groups (Fig. S9B, left). No significant difference in the phase-amplitude couplings between the HPC theta phase and the amplitude of DS high-gamma oscillations was found among groups (Fig. S9A second-row; Fig S10B right).

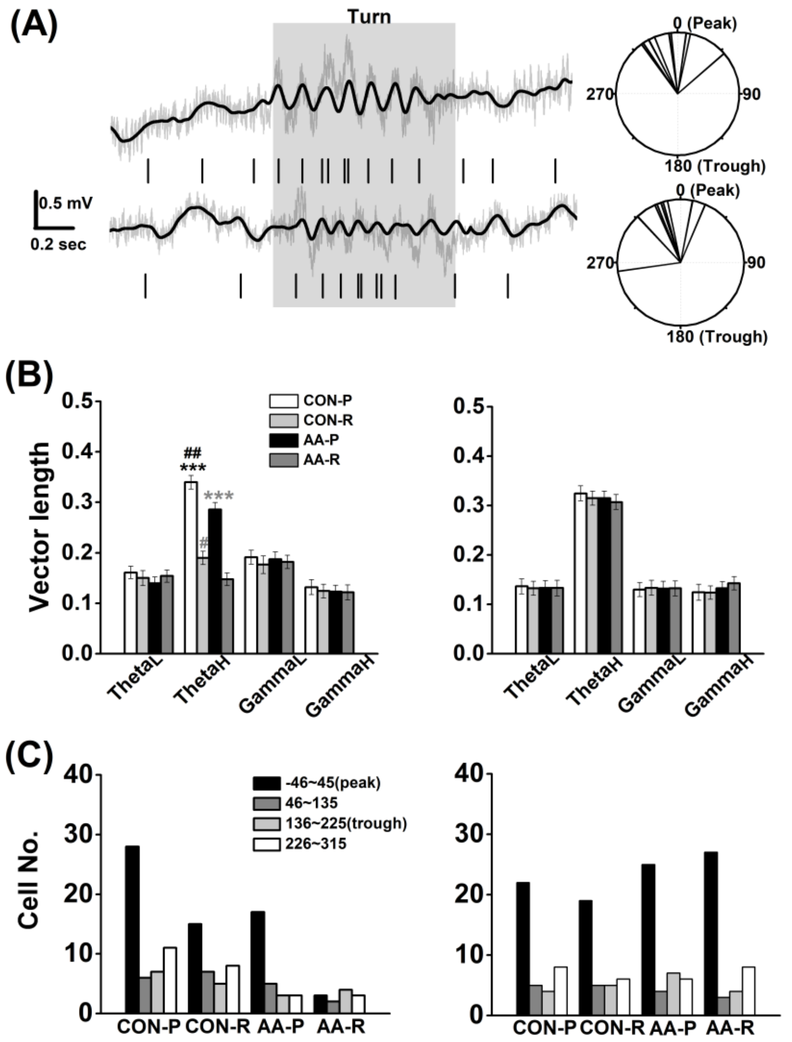

Fig. S8. Effects of AA on phase-locking between spikes and neural oscillation during strategy learning. (A) Examples of raw EEG recordings during tuning-decision period from HPC (top-left) and DS (bottom-left) overlaid with the filtered high-theta oscillation. The phase of firing of simultaneously recorded spikes from HFC (top-left) and DS (bottom-left) neurons were shown in black line, respectively. The distribution of each spike on high-theta phase in the HPC (top-right) and DS (bottom-right). The vector length from units whose firings were significant locked to high-theta oscillation **(B)** and unit number in each high-theta phase in the HPC (left) and DS (right) during turn-decision period **(C)**. (Black asterisks: ****P*<0.001, CON vs. AA-1.0; Gray asterisks: ***P*<0.01, CON vs. AA-2.0; ##*P*<0.01; black hash: CON-P vs. AA-P.). The neuron number of each group was indicated in the 2.1 section.

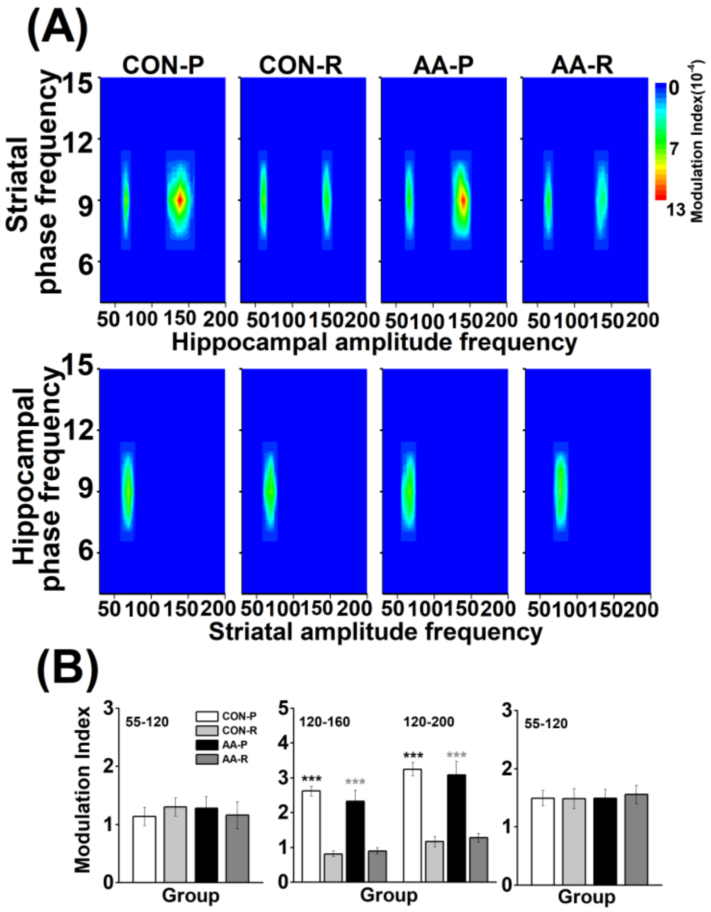

Fig. S9. Effects of AA on phase-amplitude couplings during strategy learning. (A) The phase-amplitude couplings between the DS high-theta phase and the amplitude of HPC oscillations (top) and between the HPC high-theta phase and the amplitude of DS oscillations (bottom). **(B)** The modulation index obtained from phase-to-amplitude couplings. DS theta modulation of HPC high-gamma oscillations at the range of 120-160 Hz (left). DS theta modulation of HPC high-frequency oscillations at the range of 120-160 Hz (middle-left) and 120-200 Hz (middle-right). HPC theta modulation of DS high-gamma oscillations at the range of 55-120 Hz (right). (****P*<0.001; black asterisks: CON-P vs. CON-R; gray asterisks: AA-P vs. AA-R). *n*=7 for each group.

3.3 Effect of AA on pPKA expression.

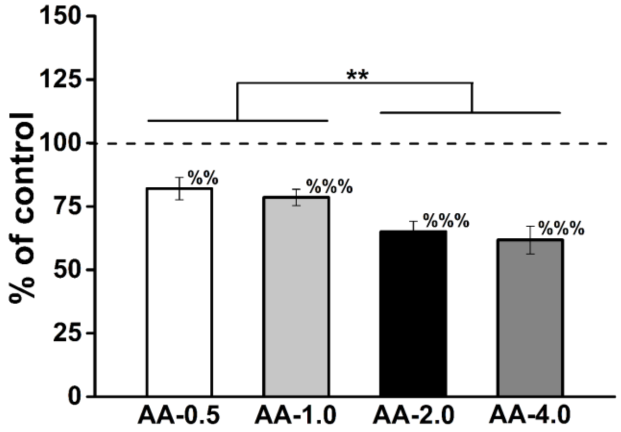

Fig. S10. Dose-dependent effect of AA on hippocampal pPKA expression.

Rats were intra-peritoneal injected with alcohol (0.5, 1.0, 2.0, or 4.0 g/kg) and hippocampus was collected after 30 min. pPKA expression was decreased than basal level (100%) following ethanol exposure (paired t-test, $P<0.01$ for 0.5 dose; $P<0.001$ for other doses). Note that 2.0 and 4.0 dose of alcohol induced a more reduction in pPKA level than lower dose of 0.5 and 1.0. Data are shown as mean ± SEM. $n=6$ for each group.

Table S1

Group	Firing rate		HPC LFP power (mv^2/Hz)		HPC Vector length	
	HPC	DS	$theta_H$	$Gamma_H$ ($\times10^{-3}$)	$theta_H$	$gamma_H$
CONP	0.88±0.11	0.72±0.10	0.34±0.02	7.6±0.6	0.34±0.01	0.13±0.02
CONP-Sp 0.21	0.86±0.16	0.69±0.12	0.33±0.03	6.8±0.5	0.32±0.02	0.13±0.01

Table S1. The data are represented as the mean firing rate of hippocampal pyramidal neurons and striatal MSNs. n = 52 for hippocampal pyramidal neurons of CONP; n = 39 for striatal MSNs of CONP. n = 55 for hippocampal pyramidal neurons of CONP-Sp0.21; n = 46 for striatal MSNs of CONP-Sp0.21. HPC LFP power is represented as hippocampal LFP power in high-theta and

high-gamma bands. *n* = 7 for each group. HPC Vector length is hippocampal vector length in high-theta and high-gamma bands. *n* = 52 for hippocampal pyramidal neurons of CONP; *n* = 39 for striatal MSNs of CONP. *n* = 55 for hippocampal pyramidal neurons of CONP-Sp0.21; *n* = 46 for striatal MSNs of CONP-Sp0.21. Infusion of Sp-cAMP into the HPC did not affect neural activity of place strategy rats. All data are presented as mean ± S.E.M..

References

1. Stark E, Roux L, Eichler R, Senzai Y, Royer S, Buzsaki G. Pyramidal cell-interneuron interactions underlie hippocampal ripple oscillations. *Neuron* (2014) 83(2):467-80.
2. Stark E, Roux L, Eichler R, Buzsaki G. Local generation of multineuronal spike sequences in the hippocampal CA1 region. *Proc Natl Acad Sci U S A* (2015) 112(33):10521-6.
3. van Wingerden M, Vinck M, Lankelma JV, Pennartz CM. Learning-associated gamma-band phase-locking of action-outcome selective neurons in orbitofrontal cortex. *J Neurosci* (2010) 30(30):10025-38.
4. Ros-Simo C, Moscoso-Castro M, Ruiz-Medina J, Ros J, Valverde O. Memory impairment and hippocampus specific protein oxidation induced by ethanol intake and 3, 4-methylenedioxymethamphetamine (MDMA) in mice. *J Neurochem* (2013) 125(5):736-46.
5. Givens BS, Breese GR. Electrophysiological evidence that ethanol alters function of medial septal area without affecting lateral septal function. *J Pharmacol Exp Ther* (1990) 253(1):95-103.
6. Tort AB, Kramer MA, Thorn C, Gibson DJ, Kubota Y, Graybiel AM, et al. Dynamic cross-frequency couplings of local field potential oscillations in rat striatum and hippocampus during performance of a T-maze task. *Proc Natl Acad Sci U S A* (2008) 105(51):20517-22.
7. Daume J, Gruber T, Engel AK, Friese U. Phase-Amplitude Coupling and Long-Range Phase Synchronization Reveal Frontotemporal Interactions during Visual Working Memory. *J Neurosci* (2017) 37(2):313-22.
8. Scheffer-Teixeira R, Belchior H, Caixeta FV, Souza BC, Ribeiro S, Tort AB. Theta phase modulates multiple layer-specific oscillations in the CA1 region. *Cereb Cortex* (2012) 22(10):2404-14.
